# Plant and animal endemism in the eastern Andean slope: challenges to conservation

**DOI:** 10.1186/1472-6785-12-1

**Published:** 2012-01-27

**Authors:** Jennifer J Swenson, Bruce E Young, Stephan Beck, Pat Comer, Jesús H Córdova, Jessica Dyson, Dirk Embert, Filomeno Encarnación, Wanderley Ferreira, Irma Franke, Dennis Grossman, Pilar Hernandez, Sebastian K Herzog, Carmen Josse, Gonzalo Navarro, Víctor Pacheco, Bruce A Stein, Martín Timaná, Antonio Tovar, Carolina Tovar, Julieta Vargas, Carlos M Zambrana-Torrelio

**Affiliations:** 1 NatureServe, 4600 North Fairfax Drive, Floor 7, Arlington, VA 22203, USA; 2 Herbario Nacional de Bolivia, Universidad Mayor de San Andrés, La Paz, Bolivia; 3 Museo de Historia Natural, Universidad Nacional Mayor de San Marcos, Apartado 140434, Lima-14, Perú; 4 Fundación Amigos de la Naturaleza, km 7,5 Doble Vía la Guardia, Santa Cruz de la Sierra, Bolivia, Casilla 2241; 5 Instituto de Investigaciones de la Amazonía Peruana, Iquitos, Perú; 6 Rumbol, S.R.L. Av. Dorbigni 1608, Cochabamba, Bolivia; 7 Asociación Armonía, BirdLife Internacional, Avenida Lomas de Arena 400, Casilla 3566, Santa Cruz de la Sierra, Bolivia; 8 Centro de Datos para la Conservación, Departamento de Manejo Forestal, Facultad de Ciencias Forestales, Universidad Nacional Agraria La Molina, Apartado 456, Lima 100, Perú; 9 Museo Nacional de Historial Natural, Colección Boliviana de Fauna, Casilla 8706, La Paz, Bolivia; 10 Nicholas School of the Environment, Duke University, Box 90328, Durham, NC 27708, USA; 11 The Nature Conservancy, 99 Bedford St., 5th Floor, Boston MA 02111 USA; 12 The Nature Conservancy, 4245 Fairfax Drive, Arlington, VA 22203 USA; 13 Ontario Ministry of Natural Resources, 50 Bloomington Road W, Aurora, ON L4G 3G8; 14 National Wildlife Federation, 901 E Street, NW Suite 400, Washington DC, 20004 USA; 15 Departamento de Ciencias, Pontificia Universidad Católica del Perú, Av. Universitaria 1801, Lima 32, Peru; 16 EcoHealth Alliance - 460 W 34th Street, 17th Floor, New York, NY 10001, USA

**Keywords:** Andes-Amazon, conservation planning, ecological systems, endemic species richness, irreplaceability, Latin America

## Abstract

**Background:**

The Andes-Amazon basin of Peru and Bolivia is one of the most data-poor, biologically rich, and rapidly changing areas of the world. Conservation scientists agree that this area hosts extremely high endemism, perhaps the highest in the world, yet we know little about the geographic distributions of these species and ecosystems within country boundaries. To address this need, we have developed conservation data on endemic biodiversity (~800 species of birds, mammals, amphibians, and plants) and terrestrial ecological systems (~90; groups of vegetation communities resulting from the action of ecological processes, substrates, and/or environmental gradients) with which we conduct a fine scale conservation prioritization across the Amazon watershed of Peru and Bolivia. We modelled the geographic distributions of 435 endemic plants and all 347 endemic vertebrate species, from existing museum and herbaria specimens at a regional conservation practitioner's scale (1:250,000-1:1,000,000), based on the best available tools and geographic data. We mapped ecological systems, endemic species concentrations, and irreplaceable areas with respect to national level protected areas.

**Results:**

We found that sizes of endemic species distributions ranged widely (< 20 km^2 ^to > 200,000 km^2^) across the study area. Bird and mammal endemic species richness was greatest within a narrow 2500-3000 m elevation band along the length of the Andes Mountains. Endemic amphibian richness was highest at 1000-1500 m elevation and concentrated in the southern half of the study area. Geographical distribution of plant endemism was highly taxon-dependent. Irreplaceable areas, defined as locations with the highest number of species with narrow ranges, overlapped slightly with areas of high endemism, yet generally exhibited unique patterns across the study area by species group. We found that many endemic species and ecological systems are lacking national-level protection; a third of endemic species have distributions completely outside of national protected areas. Protected areas cover only 20% of areas of high endemism and 20% of irreplaceable areas. Almost 40% of the 91 ecological systems are in serious need of protection (= < 2% of their ranges protected).

**Conclusions:**

We identify for the first time, areas of high endemic species concentrations and high irreplaceability that have only been roughly indicated in the past at the continental scale. We conclude that new complementary protected areas are needed to safeguard these endemics and ecosystems. An expansion in protected areas will be challenged by geographically isolated micro-endemics, varied endemic patterns among taxa, increasing deforestation, resource extraction, and changes in climate. Relying on pre-existing collections, publically accessible datasets and tools, this working framework is exportable to other regions plagued by incomplete conservation data.

## Background

**Figure 1:**
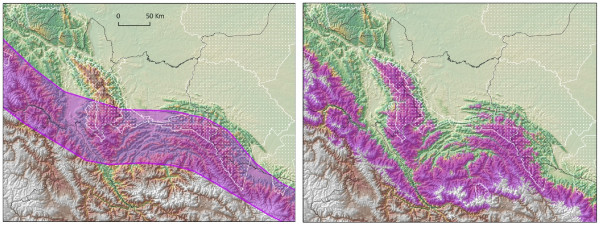
**Comparison of hand drawn vs. modelled species distribution map**. Hand-drawn range map (a) used in many continental studies with (b), a modeled species distribution for *Cycanolyca viridicyanus *in southwestern Peru (Vilcabamba). National protected areas (white), and department boundaries (black lines) and elevation as backdrop.

Numerous global conservation prioritization schemes have been developed that are centered on biodiversity, endemism and vulnerability (e.g. [1-5]). Characterizing global areas of high biodiversity under threat as "hotspots" [1] or "priority ecoregions" [6], for example, has identified priorities using a variety of weighting schemes (e.g. [3,4]). However, the information that underlies these prioritizations in the best cases can consist of coarse scale species range maps, typically hand-drawn by knowledgeable researchers from available locality data [7-10]. In less than ideal cases, lists of known species by large areal units such as ecoregions are used [11]. Although the range maps are convenient accompaniments for species accounts in field guides, they are too coarse for landscape-level conservation planning (Figure 1). There are often errors in the locality information that is used to generalize range maps, and they typically overestimate areas of occupancy because of the coarse scale at which they are drawn [12,13]

Global prioritization areas themselves are typically too large to protect in their entirety (e.g. the Andean 'hotspot' *sensu *[1], covers an area over four times the size of Germany and crosses over seven Andean countries), and are not practical nor intended for use in national or departmental planning. For many data-poor countries however, global datasets such as these are the only consistent estimates of biodiversity that are available. Effective on-the-ground conservation efforts and decisions require planning and biodiversity information at a much finer scale [14].

Endemic species are restricted to a particular geographic area-occurring nowhere else-and are important components in most global conservation prioritizations. A focus on endemic species richness can provide unique information about biodiversity patterns [3,15] compared to all-encompassing species richness that is dominated by generalist (non-endemic) species [4], which are typically the lowest priority for conservation. Areas high in endemism are especially valuable because they may represent areas of high past speciation in evolutionary hotspots [16]. The forces that create areas of high species endemism and richness are still not well understood, which is one argument for their preservation for further study [17]. Another reason for preservation is that these areas may function as species refugia during future climate changes, as they may have in the past. Globally, areas of high endemism are currently underrepresented by the protected area network [2].

The Andes region of South America harbours one of the largest assemblages of endemic plant and animal species and is one of the most biodiverse and threatened areas of the world [1-5]. Explanations for such a concentration of endemics include past climate shifts, geotectonic events, modern ecological interactions, and limited dispersal. This area was historically isolated from the lowlands by the Andean uplift, which created a complex mosaic of high mountains and deep inter-Andean valleys. Researchers generally agree that this ancient uplift and isolation were important drivers in speciation, resulting in high concentrations of endemic birds [18-22], mammals [23], and plants [24-27]. Analyses of Andean amphibians are limited but indicate similar drivers of environmental divergence [28-30] and colonization from different regions [31]. Recent climatic stability influenced by topography has created ideal conditions for high biodiversity (very humid areas) and endemism (dissected topography creating isolated dry valleys) [32].

Despite the agreement among scientists about the origins and existence of the extremely high endemic diversity of this region, it remains scientifically understudied [33]. We have very limited knowledge of current patterns of Andean species distributions and diversity within this globally prioritized area [14]. National-level efforts to prioritize conservation in Peru and Bolivia have previously explored gaps in protected area coverage, but have been hindered by the limited information available on species status and distribution [34,35]. The information available is primarily of bird diversity patterns rather than other taxon groups [36-40]. Yet even the most recent endemism studies of birds were delimited by a 1/4° grid (~28 × 28 km) as the unit of analysis [36,37]. Studies of the spatial pattern of Andean endemic mammal richness are lacking, possibly due to unstable taxonomy and incomplete knowledge about distributions [41]. A worldwide distributional analysis at a coarse scale with a 1° grid (~111 × 111-km) showed a relative concentration of endemic mammal species along the east side of the Andes in Peru and northern Bolivia [5]. As well, a regional study in Peru corroborated this pattern [42]. We are unaware of spatially explicit analyses of amphibian endemic patterns, although several authors have suggested that higher concentrations of endemics should be found in montane regions [43-45]. Knowledge of endemic plants in this region varies widely by taxonomic group. Analyses of a few better-known groups suggest peaks of diversity and endemism in the eastern Andes [17,46-49]. Vegetation and land cover maps of this region have variable coarse spatial and classification detail; different regions employ distinct classification schemes and methods that make joining maps along borders difficult.

The development of computer-aided models to predict species distributions presents an opportunity to develop distribution information at the scale necessary for in-country conservation planning [50,51]. With the goal of producing relatively fine resolution species and ecosystem data within a repeatable framework of methods, we created geographic distributions of endemic birds, mammals, amphibians, plants, and mapped their ecosystems on the eastern slope of the Andes in Peru and Bolivia at a scale applicable to conservation planning (1 km^2 ^grid, less than < 1/60°, 1:250,000 - 1:1,000,000). This multiple taxon approach enables a broader characterization of diversity, given that one taxonomic group or species is not always representative of other taxa [15,52,53]. By geographically integrating this data, we identify areas of high endemic concentrations and irreplaceable areas (greatest number of narrowly distributed endemics) across the study area [54]. We characterize the ecological systems where endemic species reside and perform a gap analysis to identify species ranges, endemic concentrations and ecological systems currently located outside of established national-level protected areas. In addition to pinpointing candidate areas for future protection efforts, the results highlight several challenges to conservation in the region.

## Methods

In addition to the following descriptions of endemic distribution modelling, mapping of ecological systems and geographical analysis of all the overlapping datasets, the Supporting Information Additional Files 1, 2, 3, 4, 5, 6, contain further method details.

### Study Area

**Figure 2:**
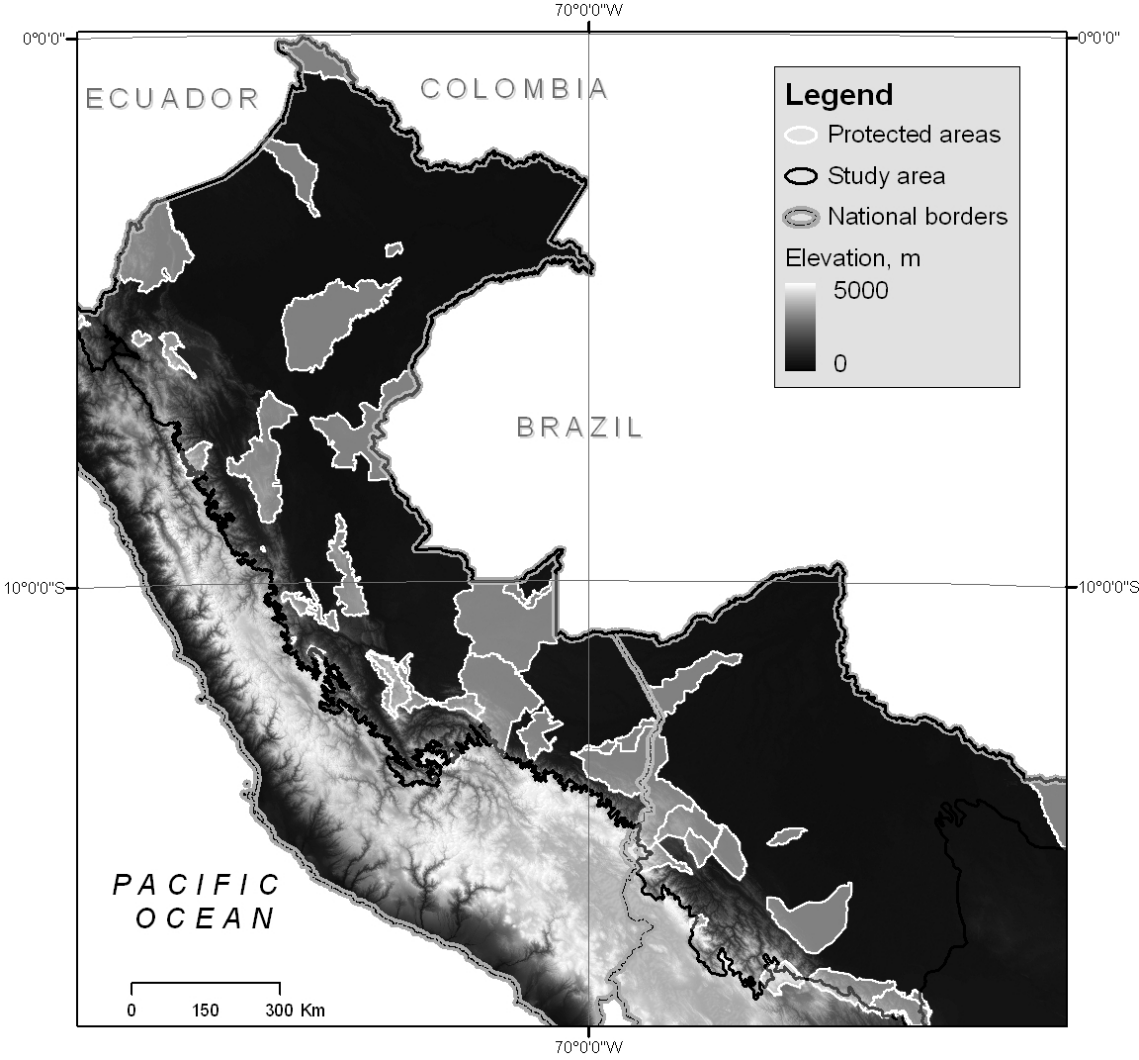
**Study area**. The Amazon basin of Peru and Bolivia with current protected areas. Protected areas information from INRENA-Peru, and FAN-Bolivia, elevation from Shuttle Radar Topography Mission.

Our study focused on the Amazon basin of Peru and Bolivia, from treeline in the eastern Andes (~3500 m), downslope to the Amazon lowlands and extending to the Brazilian border (Figure 2). The southern limit extends to the edge of the southern subtropical uplands where the biogeographic province of the *chiquitanía *begins. The area hosts a wide range of ecosystems from the wetlands of the Beni savanna and the Iquitos *várzea*, to xeric habitats of inter-Andean valleys and humid montane forests along much of the eastern Andean slope. Many areas are difficult to access because of lack of transportation infrastructure, entrance restrictions into indigenous lands and patrolling of illegal crops [36]. The study area extends from 5°23' to 18° 15' S latitude and from 60° 23' to 79° 26' W longitude and covers 1,249,282 km^2^.

### Endemic Species and Locality Data

More than a century of collecting in South America has yielded large numbers of plant and animal specimens that provide locality data for species geographic distribution predictions. To represent a diverse suite of species, we modelled the geographic distributions of all bird, mammal, and amphibian species that are endemic to our study area [7-9] (Table 1). We identified which species were endemic based on pre-existing hand drawn range maps [8,9,55] and consultation with regional experts. We also modelled distributions of endemic plants but limited our analysis to 15 representative focal groups (families or genera) generally well known, and relatively well sampled in both countries (details on criteria for inclusion can be found in Additional File 1): Acanthaceae, Anacardiaceae, Aquifoliaceae, Brunelliaceae, Campanulaceae, Chrysobalanaceae, Cyatheaceae, Ericaceae, *Inga *(Fabaceae), *Mimosa *(Fabaceae), Loasaceae, Malpighiaceae, Marcgraviaceae, *Fuchsia *(Onagraceae), and Passiflora (Passifloraceae). As with the vertebrates, we modelled distributions for all species in these groups that are endemic to the study area.

**Table 1:** Summary of endemic species groups and modeled ranges

Species group	Number species	Number genera	Total number localities	Median number records per species	No. data sources collaborating Institutions	Number Maxent models formed	Median distributional area, (km^2^)
*Amphibians*	177	30	1060	2	9	85	399
*Birds*	115	69	2437	15	15	99	21,075
*Mammals*	55	29	618	7	12	47	24,156
*Plants*	435	66	3040	3	50+	264	3543

For each of the 782 species of endemic plants and animals, we compiled locality records from an exhaustive search of specimen records in 81 local and international natural history collections and herbaria, published records, and for birds and mammals only, observational data. Specimen searches were carried out 2004-2006 with Peruvian, Bolivian and international institutions, individuals, and from published sources (see Additional File 5). The majority of specimens were collected in the 1990's and 2000's, yet dates ranged wider for published sources that we validated with national gazetteers of collecting locations [56]. The oldest localities for example, were collected for mammal species in the early part of this century [57]. Because many specimen labels did not include global positioning system-based coordinates for the collecting locations, we identified the most reliable localities based on their described location and geo-referenced them using standardized methods [58], and additional resources such as consultation with the collector, and geographic gazetteers (e.g.[56]). To further assure the creation of an accurate locality database, we then asked taxonomic specialists familiar with the species and geography to review mapped localities to ensure the creation of an accurate locality database. We buffered the study area by 100 km for the endemic species data gathering and modelling to avoid edge effects.

### Predictive Distribution Modelling

We used spatial environmental layers describing climate, topography, and vegetation within our study area at 1-km^2 ^resolution together with the field locality data to develop species distribution models (Table 2). The WorldClim climate data [59] is currently the best available for this region yet it has its own inaccuracies as will any future downscaled version, because meteorological information is scarce in many areas of the study area. To maintain consistent spatial resolutions, we resampled the most accurate elevation data for the region (NASA's Shuttle Radar Topography Mission, SRTM [60]) to match the 1-km^2 ^resolution of the climate data. Vegetation characterizations were made with a 3-year seasonal time series of satellite-derived MODIS vegetation indices and per cent tree cover [61] at the same resolution. Mapping of detailed ecological systems, discussed below, was conducted separately as an independent characterization at a higher spatial resolution based on NASA's Landsat Thematic Mapper satellite sensors.

**Table 2:** Environmental predictors and data sources for species distribution modelling

*Variable*	*Data Source*
Mean annual temperature, mean temperature diurnal range, isothermality, precipitation of wettest and driest month, precipitation seasonality	Worldclim, (Hijmans et al. 2005. www.worldclim.org), 1-km resolution
Topography: Elevation	Shuttle Radar Topography Mission digital elevation data provided by CGIAR (http://srtm.csi.cgiar.org/) resampled to 1-km resolution
Slope	Degree of slope (maximum rate of change in elevation from each pixel to its 8 neighbors) derived from the SRTM digital elevation data
Topographic exposure	Expresses the relative position of each pixel on a hillslope (e.g. ridge, valley, toe slope). Using methods of Zimmermann (2000) on the SRTM digital elevation data with three neighborhood windows of 3x3, 6x6 and 9x9
Percent tree cover	MODIS global vegetation continuous fields sourced from http://glcf.umiacs.umd.edu/data/modis/vcf/data.shtml (Hansen et al. 2003) 1-km resolution, and summarized within 3- and 5- km moving windows
Enhanced Vegetation Index (EVI) Principal component 1 Principal component 2	MODIS vegetation indices 16-Day data product sourced from the NASA EOS data gateway; Principal component analysis of 3 years of 16-day composites. MODIS EVI data summarized within 5 km moving window

There are drawbacks to predictive distribution modelling-for example, models may overestimate species' geographic ranges [62,63]-as well as advantages, such as reducing the effect of uneven collecting efforts [64]. Nonetheless, distribution modelling is arguably the best approach at present when reliable locality and environment data are available [65]. We chose Maximum Entropy ("Maxent") [66], a statistical mechanics approach, as our modelling algorithm because of its documented success at modelling species with limited locality data, a common problem when working with endemic species [65,67-69]. To ensure that Maxent was best suited to modeling distributions of Andean species, we compared the success of Maxent and two new promising methods: Mahalanobis Typicalities (a method adopted from remote sensing analyses), and Random Forests (a model averaging approach to classification and regression trees). We found that Maxent produced more consistent predictions across varying climatic conditions for 16 species [67]. Two to seven taxonomic specialists reviewed each model output to determine thresholds to convert continuous predictions into presence-absence maps based on known areas of absence, and to remove areas of known over-prediction (i.e., where the species was known not to occur). Specialist review is especially necessary when modelling with small sets of locality data [52,67,70]. For species known from a single or very few localities, we ran "rule-based" models (instead of Maxent) consisting of the geographic intersection of known ranges in elevation and other environmental variables such as temperature and precipitation.

### Areas of Endemism and Irreplaceability

Traditionally, ecologists have overlain distribution maps of species to identify areas of high endemism or species richness [39]. We followed this approach to identify areas of high endemism for each vertebrate and plant group. To identify discrete areas of high endemism we chose an arbitrary threshold value of two-thirds the maximum number of overlapping species for each group and compared these patterns with previous studies, where they exist. This simple threshold could be changed depending on the desire to be more or less inclusive in identifying areas of high endemism.

To highlight areas harbouring species with very restricted ranges, and therefore of potentially greater conservation significance, we created maps of summed irreplaceability for each group using the C-Plan Software [71]. Summed irreplaceability is the likelihood that a given analysis unit should be protected to achieve a specified conservation target for the study area [54]. We used 10-km^2 ^analysis pixels and defined 25 of these pixels for each species as a conservation "target". If a given species was found present in < 25 of the 10-km^2 ^pixels, we set the target as the number of pixels in which the species occurs. For each species, irreplaceability for each pixel ranges from 0 to 1. Low values of irreplaceability indicate that for a species there are many other (replaceable) sites that may be conserved (in other words that a species occurs in many pixels), whereas high values indicate there are very few sites available (irreplaceable) because the species have very narrow ranges. The final irreplaceability number is the result of summing irreplaceability values for all species occurring at each location, thereby emphasizing the locations with the higher number of narrow-range endemics.

### Ecological Systems

To complement the endemic species information, we produced a detailed map of natural vegetation types at a scale of 1:250,000 (25 ha minimum mapping unit). We applied a hemisphere-wide vegetation classification system [72] that is the terrestrial classification employed as a standard in North America in U.S. federal mapping projects [73,74] and an emerging standard in Latin America [75]. The classification relies on the concept of terrestrial ecological systems [73], which are groups of vegetation communities that tend to co-occur in landscapes as a result of the action of common ecological processes, substrates, and/or environmental gradients. The ecological system classification allows for effective integrated vegetation mapping, at desired levels of thematic detail, permitting planners to prioritize across borders and across large regions. The species distribution models did not use this map as a predictor variable, thus the map provides an independent characterization of areas where endemics reside. In addition to analysing protection gaps and representativeness of the systems, we examined the overlap between ecological systems and areas of high endemism. Our goal was to identify if any systems were disproportionally represented in endemic areas compared to their distributions across the study area.

To create the ecological systems map, we incorporated existing vegetation maps where possible, and with in-country mapping teams of local field and botanical experts; we applied one cohesive classification system across the two countries. The mapping relied on field work, visual interpretation of Landsat TM and ETM+ satellite images in the Peruvian lowlands and areas of Bolivia, and spatial modelling and image classification for upland areas in Peru. Though more advanced mapping methods exist (e.g., [76]), we found our methods to be appropriate for these landscapes and the limited data availability, as well as more accessible to the in-country mapping teams. For ecological system characterization as well as accuracy assessment, we developed a rapid field survey protocol for more than 2000 points across the study area using spatial optimization to identify candidate clusters of points. Field observations and aerial transects of high-resolution digital photos of remote and inaccessible areas provided the basis for map validation and accuracy assessment. Details of the mapping methods, classification system and accuracy assessment can be found in Additional File 1.

### Gap Analysis

We conducted a gap analysis (*sensu *[77]) by examining the representation of terrestrial ecological systems, species distributions, and areas of high endemism and irreplaceability with respect to existing national-level protected areas. We included all designated nationally administered areas corresponding to World Conservation Union (IUCN) categories I-VI (IUCN 1994), as well as those that have not yet been scored against the IUCN criteria. This covered national parks, communal reserves, protected forests, integrated management areas, and other national sanctuaries. Rather than limiting our analysis to those areas with IUCN categories reflecting the strictest levels of protection, we took an inclusive approach, recognizing that in this region effective protection can vary in any category. We used digital maps of protected area boundaries from 2007 provided by our in-country collaborators as they were more current than the World Database of Protected Areas WDPA [78] at the time. National level protected area boundaries have not changed in the region at the time of publication of this article; however improvements have been made to the WDPA information. Regional protected areas have experienced shifts in jurisdiction, area, and level of protection. While including regional protected areas in this analysis would be advantageous, information on protection levels and boundaries of regional areas is incomplete in some areas and inconsistent across country borders.

## Results

The datasets and individual species maps for most of the analyses described here are publically accessible (in both graphic and geospatial format) on the project website (http://www.natureserve.org/andesamazon). The supporting Additional Files 1, 2, 3, 4, 5, 6, contain supplementary results in detail.

### Endemic Species

**Figure 3:**
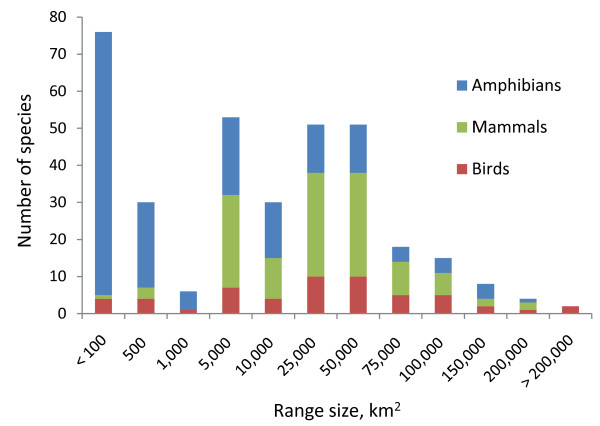
Distribution of predicted range sizes by species groups

We compiled 7154 unique records of existing specimen localities to create distribution models for all 115 birds, 55 mammals, 177 amphibians, and 435 plants included in our endemic species analysis (Table 1; see Additional File 1). Sample sizes of unique localities for modelling distributions of individual species were highest for birds, followed by mammals, plants, and amphibians. There were 3 mammal and 3 bird species having just one reliable locality, whereas 123 plant and 65 amphibian species were limited to one location, none of which were predicted with distribution modelling. Modelled distribution sizes varied from just 2 km^2 ^for the plant *Centropogon bangii*, to 690,992 km^2^, or 55% of the study area, for the frog *Colostethus trilineatus*. On average, endemic mammals tended to have the largest geographic distributions, followed by birds, plants and amphibians (Figure 3). Maxent models produced satisfactory distribution maps, according to expert reviewers and model evaluation techniques, for 67% of the species. We produced distributions for the remaining species, which had too few known localities for Maxent models, using rule-based models. Expert review was essential for eliminating areas from the distribution where the species was known not to occur for reasons of competition or geographic isolation.

### Areas of Endemism and Irreplaceability

**Figure 4:**
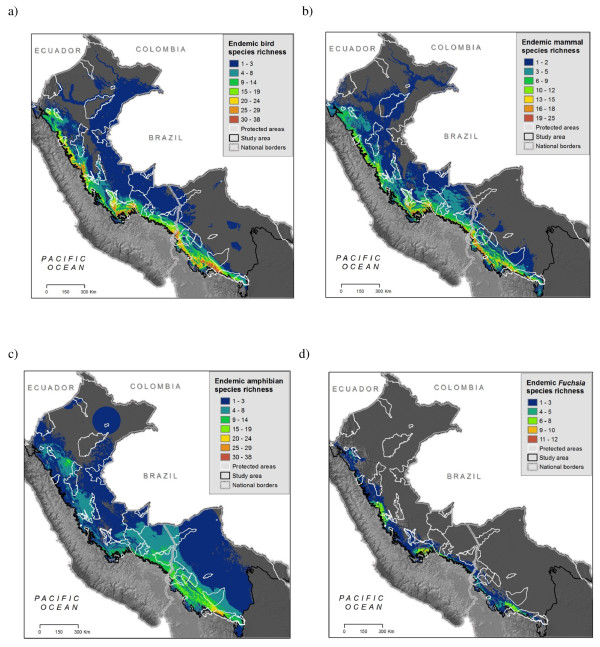
**a-d - Endemic species richness**. Overlapping distribution maps for different species groups: a) birds, b) mammals c) amphibians d) Fuchsia genus plant species. Fuchsia is shown as an example of one of the 15 groups modelled (See http://www.natureserve.org/andesamazon for maps of individual species and all plant groups).

**Figure 5:**
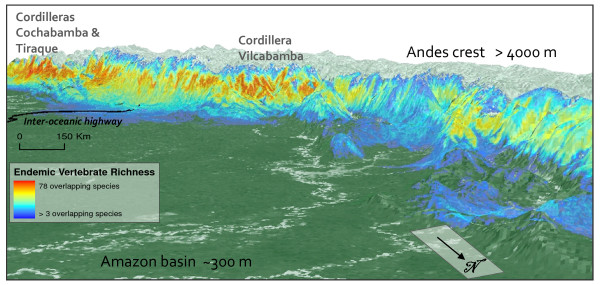
**Endemic vertebrate species richness**. Combined endemic mammal, bird, and amphibian richness over a three-dimensional oblique perspective. Viewpoint is from northeastern Peru looking south across the Amazon basin towards the southern Peruvian and northern Bolivian Andes.

Areas with the highest numbers of endemic species lie along mid to upper elevations on the eastern slope of the Andes, yet patterns vary by taxonomic group. Both birds (25-38 species per 1-km^2 ^grid cell) and mammals (17 - 20 species per cell) followed this trend (Figures 4a, b) with peaks of endemic richness encompassing elevations between 2500 and 3000 m and extending almost the entire length of the study area. Amphibians, by contrast, displayed peaks of endemism (21 - 29 species per 1-km^2 ^cell) on lower slopes, between 1000 and 1500 m elevation. These areas were concentrated in southern Peru, northern Bolivia, and in an isolated endemic area in the northern Peruvian department of San Martin (Figure 4c). Combining all vertebrate species reveals high concentrations between 2000 and 3000 m elevation (Figure 5) with highest concentrations (75 to 78 overlapping species) in Bolivia's Cochabamba and Tiraque *Cordilleras *(mountain ranges) and extensive areas of high value along Peru's Vilcabamba Cordillera. We found that the different plant groups varied widely in endemic patterns among themselves and with respect to vertebrates. Areas of high *Fuchsia *endemism, for example, were at similar elevations as birds and mammals, but with local concentrations in the departments of Cusco (Peru), and Cochabamba (Bolivia) (Figure 4d). Endemic species of Aquifoliaceae, Chrysobalanaceae, *Inga*, Loasaceae, and Malpighiaceae were concentrated in the northern portion of the study area, whereas endemic Brunelliaceae, Campanulaceae, Ericaceae, Marcgraviaceae, *Mimosa*, and Passifloraceae were concentrated in the south. We found concentrations of endemic Acanthaceae in both the north and south. Endemic species of Anacardiaceae, Chrysobalanaceae, *Inga*, and Malpighiaceae were concentrated in the lowlands, whereas Acanthaceae and Cyatheaceae occurred largely at mid elevations (around 1000 m); endemic species in the remaining nine groups occur mostly above 2000 m (maps of all plant species can be found here: http://www.natureserve.org/aboutUs/latinamerica/maps_plants_intro.jsp).

**Figures 6:**
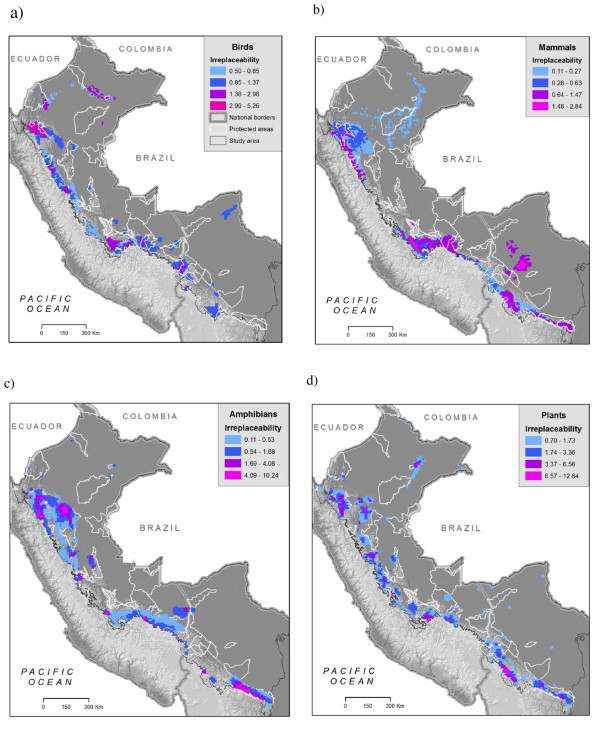
**a-d - Summed irreplaceability analysis for different species groups**; a) birds, b) mammals c) amphibians d) plants from all 13 groups.

Summed irreplaceability analysis which highlights areas with the greatest numbers of narrow-ranging species, shows different key areas than the endemic areas analysis. Similar to the endemic areas, many of the peaks of summed irreplaceability occurred in the higher elevation slopes along the Andean cordillera (Figure 6a-d, areas over threshold value shown). Endemic richness of birds and mammals overlapped more than other groups yet summed irreplaceability showed differences between these two taxonomic groups, as well as for amphibians. The northern portion of the study area in the Peruvian department of Amazonas (Cordillera de Colán and Alto Mayo) is highly irreplaceable for plants, amphibians, and birds but was not identified as an endemic area by the simple overlay of species ranges (Figures 4a, c, d); this emphasizes the large number of very restricted range species that occur there. Summed irreplaceability also highlighted some lowland areas for species groups in which most other species occurred at higher elevations. For instance, birds have high irreplaceability in north-eastern Peru, where a number of species are restricted to the lowland white-sand forests near Iquitos. Similarly, there are two restricted range primate species in the Beni savanna of Bolivia, emphasizing the irreplaceability of that region for mammals. Detailed descriptions of locations of the areas of high endemism and irreplaceability for all species groups can be found in Young et al. (2007).

**Figure 7:**
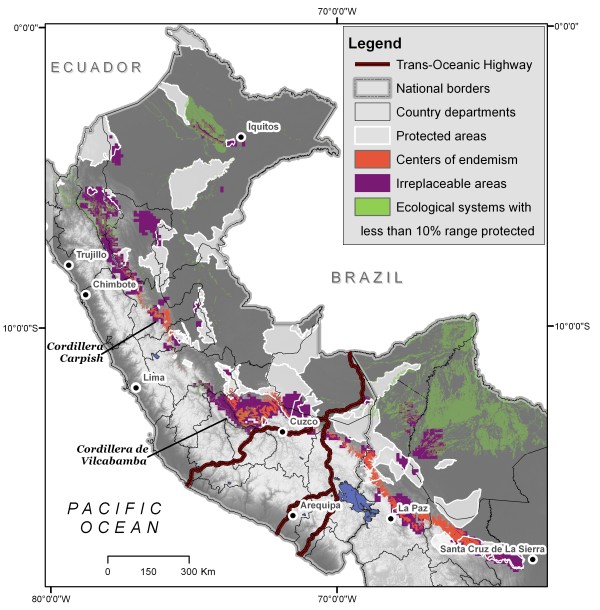
**Biodiversity indicators**. Discrete centres of vertebrate endemism and high levels of summed irreplaceability (all species groups), and ecological systems with less than 10% of their ranges protected.

Discrete centres of endemism (Figure 7), covered 23,844 km^2 ^for birds, 11,655 km^2 ^for mammals, 2781 km^2 ^for amphibians, and 67,676 km^2 ^for plants. (We only included 13 groups for plants as Anacardiaceae and Cyatheaceae did not have more than two co-occurring endemic species anywhere in the study area.) Combining all plant and animal endemic areas results in a region covering 78,790 km^2 ^or 6.3% of the study area. In contrast, the intersection of endemic areas for the three vertebrate groups covers a mere 140 km^2^, highlighting differences among these groups.

### Ecological Systems

**Figure 8:**
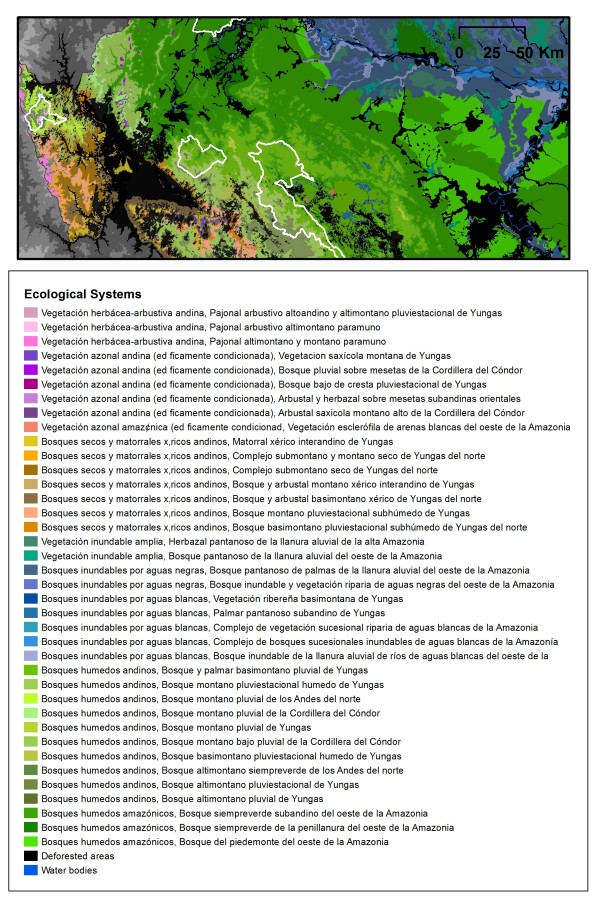
**Ecological systems detail of subarea in northern Peru**.

We distinguished 91 unique ecological systems and complexes across the basin, ranging from flooded savanna systems to xeric shrub types (Figure 8 shows an area in detail for northern Peru; see [79] for a description of each ecological system). The systems represent unique vegetation communities, further distinguished by bioclimate, geomorphology, substrate, flooding regime, river type (black, white, mixed water) and regional compositional differences. Half of the ecological systems consist of different forms of wetlands and cover 30% of the study area and systems with bamboo-dominated forests cover over 71,500 km^2^. Forty-two of the ecological systems are unique to the Amazon basin of Peru and Bolivia. The Andean uplands region of Peru, typified by steep elevation gradients and subsequent vegetation zonation [80], represents only 12% of the study area yet harbours 37% of the different ecological systems. Accuracy of the ecological system map varied by region and the type of validation data. For detailed classes of ecological systems (not including areas converted to human uses), accuracy ranged from 62 to 91% by mapping region, while a map legend of 20 coarser groupings of systems defined by ecological similarity had accuracies ranging from 81 - 90% (See Additional File 1 and 4).

Combining the areas of endemism of the three vertebrate groups creates a region covered by 16 ecological systems (Table 3). The montane pluvial forest of the *Yungas *(montane and cloud forests of the Andean-Amazon slope in Peru and Bolivia) covers ~36% of the vertebrate endemic area, yet only makes up 1.7% of the study area. This ecosystem together with the three other Yungas forest types, (lower mountain pluvial forest, montane humid pluviseasonal, upper montane pluvial forests) cover an overwhelming proportion, (77%) of the vertebrate endemic areas but themselves cover just 7% of the study area. Ecological systems that occurred in highly irreplaceable areas, were more evenly distributed in terms of system type (Table 3 second column); western Amazon sub-Andean evergreen forest covered the highest percentage (12.6%) of highly irreplaceably areas followed by the Yungas lower mountain pluvial forest (9.3%).

**Table 3:** Ecological systems that overlap vertebrate endemic areas and irreplaceable areas.

Ecological system	Percent of endemic area covered by system	Percent of irreplaceable areas covered by system	Percent of study area covered by system	Percent of system range that is protected
Montane pluvial forest of the Yungas	35.7	1.3	1.7	34
Lower montane pluvial forest and palm grove of the Yungas	16.1	9.3	3.5	41
Montane humid pluviseasonal forest of the Yungas	14.9	6.3	1.1	13
Upper montane pluvial forest of the Yungas	10.4	1.4	0.6	22
Upper montane pluviseasonal forest of the Yungas	5.2	2.6	0.6	9
Converted lands	4.6	14.7	6.0	4
Low montane subhumid pluviseasonal forest of the southern Yungas	3.3	0.8	0.6	16
Lower montane humid pluviseasonal forest of the Yungas	3.3	2.3	0.8	18
Southwestern Amazon subandean evergreen forest	2.8	5.2	5.9	42
High-Andean and upper montane pluvial grassland and shrubland of the Yungas	2.3	3.4	0.4	30
Western Amazon subandean evergreen forest	0.0	12.6	5.0	38
Southwestern Amazon piedmont forest	0.0	7.4	2.6	39
Southwestern Amazon subandean evergreen seasonal forest	0.0	5.6	1.7	48
Western Amazon semideciduous azonal forest	0.0	5.4	1.0	1
Lower montane humid pluviseasonal forest of the Yungas	0.0	2.3	0.8	18
Lower montane pluvial forest of the Condor Mountain Range	0.0	2.0	0.2	42

Systems shown cover at least 2% of vertebrate endemic (2,7676 km^2^) or irreplaceable areas (150,500 km^2^); ordered by coverage of endemic areas.

### Gap Analysis

**Figure 9:**
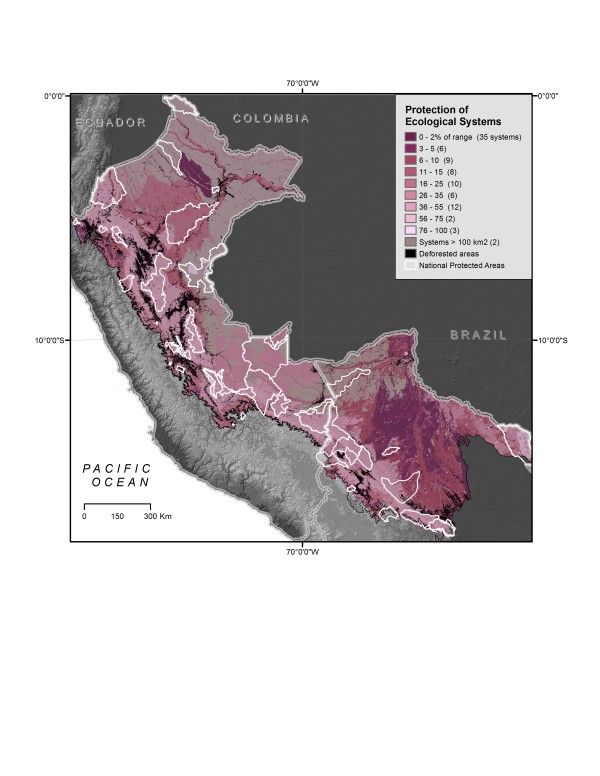
**Ecological systems protection**. Percentage of each system's range protected in study area.

National protected areas cover approximately 12% of the study area, resulting in variable levels of protection for endemic species and their ecosystems. Of the endemic species examined, 327 (42%) have less than 10% of their distributions within protected areas (Table 4 see Additional File 3). About a third of all endemic species (226) occur completely outside of protected areas. As for discrete areas of endemism, amphibian areas receive the greatest protection: 67% of the area occurs within existing protected areas. Protection for the other endemic areas was lower (birds, 7%; mammals, 29%; plants, 24%). Only 20% of the combined endemic centres occurred within national-level protected areas (Figure 7). Fewer than 20% of all combined irreplaceable areas are under national protection, with protection varying by species groups (birds, 17%; mammals, 18%; amphibians, 17%; plants, 15%) (Figures 6a-d). Five of the seventeen ecological systems that cover the areas of endemism (Table 3) have less than 5% of their extents protected across the study area. About half of the 91 ecological systems have 10% or less of their extents covered by protected areas, with 26 of these systems having less than 2% under legal protection (Table 5; Figure 9; see Additional File 3).

**Table 4:** Coverage of endemic species ranges by national-level protected areas; IUCN l - VI (IUCN, 1994)

Percent range in IUCN I-VI protected area	Birds	Mammals	Amphibians	Plants
> 75	3	3	21	15
51 to 75	5	2	11	23
26 to 50	40	23	33	97
10 to 25	44	13	29	92
< 10	23	14	83	207

No protection	5	5	72	144

**Total number of species**	115	55	177	435

**Table 5:** Terrestrial ecological systems having less than 2% protection in the study area

Ecological system	Area (ha)	Percent of study Area	Area protected (ha)	Percent protected
Complex of non-alkaline savannas of the Beni transitional to the Cerrado	2,221,743	1.8	459	0
Cerrado complex of the northern Beni	1,766,905	1.4	0	0
Western Amazon semideciduous azonal forest	1,276,552	1.0	14,533	1
Complex of non-alkaline savannas of the Beni	585,143	0.5	0	0
Central-south Amazon Palm dominated forest	578,331	0.5	0	0
Chiquitania and Beni seasonally flooded herbaceous oligotrophic savanna	506,966	0.4	0	0
Beni seasonally flooded palm grove and savanna of the alkaline flatlands	226,672	0.2	6	0
Chiquitania and Beni "Cerradão"	214,452	0.2	0	< 1
Beni seasonally flooded herbaceous mesotrophic savanna	208,539	0.2	217	< 1
Montane interandean xeric forest and shrubland of the Yungas	205,749	0.2	40	< 1
Interandean xeric scrub of the Yungas	152,396	0.1	0	0
Beni and Chiquitania open hydrophytic savanna	145,912	0.1	0	0
Lower montane xeric forest and shrubland of the northern Yungas	137,919	0.1	101	< 1
Beni mixed-water riparian vegetation and forests complex	120,637	0.1	0	0
Northern Yungas dry submontane complex	95,189	0.1	0	0
Cerrado hydrophytic savannah with termite mounds	63,280	0.1	0	0
Chiquitania and Beni semideciduous subhumid forest	48,789	< 0.1	0	0
Beni clear and dark-water riparian forests and vegetation complex	35,684	< 0.1	0	0
Central-south Amazon ridges lithomorphic scrub	21,028	< 0.1	0	0
Northern Yungas dry montane and submontane complex	19,602	< 0.1	0	0
Yungas ridge pluviseasonal forest	16,994	< 0.1	325	1
Montane lithomorphic vegetation of the Yungas	10,296	< 0.1	0	0
Western Beni seasonally flooded thorn forest of the alkaline flatlands	10,009	< 0.1	0	0
Upper montane pluvial *Polylepis *forest of the Yungas	8423	< 0.1	73	1

Several areas of endemism and irreplaceability without current national-level protected status are worth highlighting (Figure 7). In northern Peru, areas near the cities of Iquitos and Tarapoto host unique concentrations of endemic plants. The Tarapoto region also has a large irreplaceable area for amphibians. The Carpish Hills in the Department of Huanuco host many endemic plants (Acanthaceae, Aquifoliaceae and *Fuchsia*) and are highly irreplaceable for endemic birds (up to 32 ranges overlap) but are completely unprotected. The Cordillera de Vilcabamba is a major area of endemism for birds, mammals and plants (*Fuchsia*). It also constitutes the largest cohesive irreplaceable area for birds and mammals in the study area, and is highly irreplaceable for some plants. Currently the Cordillera of Vilcabamba has only one protected area, the Machu Picchu Historical Sanctuary, which covers just 326 km^2^, and is highly impacted by tourism. The north-eastern corner of the Department of Puno has numerous endemic birds and mammals and is also unprotected. However, many of the ranges of these species extend into Bolivia where they are protected in Madidi National Park.

In Bolivia, the cordilleras near La Paz have high levels of bird, mammal and plant endemism (8 of the 13 plant groups analysed), and scored as highly irreplaceable for endemic mammals and plants. Most of these cordilleras are not protected, although a small area that is irreplaceable for amphibians coincides with the 608-km^2 ^Cotapata National Park (Figure 5). In central Bolivia, unprotected endemic areas for birds, mammals, and amphibians occur in the Cordillera de Cocapata-Tiraque and Cochabamba Department, between protected areas.

## Discussion

Our results, at a conservation practitioner's scale, identify geographic areas in the eastern slopes of the Peruvian and Bolivian Andes with high concentrations of endemic species, areas with high irreplaceability, gaps in protection for both species and ecosystems, and ecological systems where these endemic species reside. Our focus on a variety of vertebrate and plant groups underlines the variation in spatial distribution patterns among different taxa. The geographical extents and levels of current protection of the ranges of species, endemic areas, irreplaceable areas, and key ecological systems also vary widely.

Mapping species distributions is inherently limited in terms of a true representation of biodiversity. As a one dimensional map of potential habitat based on climate, elevation and vegetation, the distribution modelling omits species interactions such as predation and competition, effect of human edges along habitat, and the effects of climate change [63,81]. However it is a large step forward for this region where current conservation analyses are obliged to rely upon generalized hand-drawn maps of species ranges, or species lists for very large multi-country geographical units (e.g. Hotspots or Ecoregions) that were not intended nor appropriate for regional or landscape level applications [11]. Our mapping of ecological systems, for example, identified ~90 ecological systems; the same area is covered by parts of 12 ecoregions (*sensu *[82]).

The locations of high endemism (Figure 4) agree with past studies for taxa that have been examined previously, yet earlier studies were conducted with much less data availability and at much coarser spatial resolution. The high levels of endemic bird richness found in the northern part of the study area are consistent with previous work [36,40,83]. However, our study revealed previously unrecognized areas of bird endemism in Peru: the southern Huánuco region, the western Cordillera de Vilcabamba, and the region along the Río Mapacho-Yavero east of Cuzco (Figure 4, 7; see [84] for details). This study is the first to reveal detailed patterns of endemic species for mammals and amphibians (see [85] for location descriptions), and therefore few comparisons with past studies can be made. However the areas of high endemic mammal richness in Peru corroborate the one regional study of similar scope [42] and the mid-elevation concentration of endemic amphibians coincides with the less spatially explicit suggestions of [44] and [45]. Centres of plant endemism varied among groups and families, yet the pattern for one group (*Ericaceae*) did correspond to a previous study [49]. Other existing analyses use such coarse resolution (e.g., the 1°×1° Flora Neotropica grid [47]) that comparisons are too general to be meaningful. For most plant groups, this study is the first to assess spatial patterns of endemism in the eastern Andean basin of Peru and Bolivia.

Despite the increased level of detail in spatial scale that our dataset provides, continued work needs to focus on refining these biodiversity data to even finer spatial scales (e.g. 1:100,000) and higher levels of accuracy. The dataset and analyses we have produced are tied to the time of specimen collections and to the quality of available data. As more specimen locations are collected in the future with increasingly accurate locational and elevational information (using a precise global positioning system), distribution models could be re-run and models validated. Geographical collection bias, a problem for presence-only distribution models could be addressed in future modelling efforts by the selection of pseudo-absence data having similar bias as the presence data [86]. More precise geographical climate data could refine the spatial resolution of model predictions; there will be an increasing prevalence of 'downscaled' geographical climate data thanks to higher spatial resolution digital elevation models (SRTM and ASTER). However the overall limitation is the lack of adequate meteorological stations in the region. Other layers that would be useful to incorporate upon their refinement would be a characterization of soils or geology. We successfully modelled all endemic vertebrates yet, additional models of plant species distributions should be realized. Considering there are over 5000 endemic plant species in the country of Peru (of which approximately 3200 fall within the altitudinal range of our study area) [87], our 435 species represents a small fraction of endemics to Peru and/or Bolivia in the Amazon watershed.

Our country wide analysis could be refined to department scale using land tenure information and local to regional protected areas and resource concessions. Current maps of forest deforestation and degradation would aid in calculating the remnant ranges for each species as well as ecological systems. Further analysis could be made in terms of the complementarity of species assemblages and their relationship to ecological systems and levels of protection, whose results could further guide priorities. However, the greater battle for biodiversity conservation lies in managing elements beyond our datasets and analyses, as described below.

The geographical patterns of endemism, irreplaceability, and ecosystems revealed here pose several challenges for conservation planning in the region (Figure 7). The most obvious challenge is the geographic configuration of the locations of endemic or irreplaceable areas. Although we mapped only a small subset of the biodiversity that occurs in the region, we found striking geographic differences in endemic species concentrations across taxonomic groups. The difficulty of using surrogates of one species group for another has been recognized [15,52,53], and our findings underscore the need for a large portfolio of protected areas and other protection mechanisms to conserve diverse elements of biodiversity.

Second, the gap analysis demonstrates that many areas where concentrations of endemic species occur remain unprotected today. Considering ongoing threats in the region from infrastructure development [88], oil extraction [89], gold mining [90,91], illicit crops [36], and the continually advancing agricultural fronts, more carefully situated protected areas and novel land use regulation strategies will be necessary to safeguard substantial amounts of biodiversity.

Third, although we use protected area coverage to evaluate conservation coverage, we acknowledge that protection status does not necessary translate into actual protection on the ground. Indeed, resource extraction and degradation is continuing in many legally protected lands in the study area [92]. Nevertheless, these reserves have the potential to protect important segments of endemic and irreplaceable areas, suggesting that strengthening the capacity of relevant authorities to improve protection is an important and continuing challenge.

Fourth, large reserves will probably be insufficient to maintain all biodiversity. Although large reserves often provide the best means for maintaining well-functioning ecosystems [93], the pattern of endemism we document, in which microendemic species are scattered across the landscape and not always concentrated geographically, will require multi-pronged conservation efforts. Restricted-range species that occur far from the major areas of endemism or irreplaceability, such as the two primates in the Bolivian Beni, would benefit from a wider network of smaller reserves, perhaps established by departmental, provincial, or municipal governments or private entities. Current trends toward the decentralization of responsibility for natural resource management to provincial governments may provide a useful institutional context for the establishment of some of these smaller, but nonetheless critical reserves [94].

Our finding that highly endemic areas disproportionally occupy a handful ecological systems presents yet a fifth challenge. Ecological systems characterize broad, integrated units of biodiversity and can be used as a coarse filter for conservation. While maintaining representation of all systems in landscape-level protection plans [95], planners may need to balance the need to protect endemic species with the need for a representative sample of ecosystem type and function as well as other targets such as endangered species or carbon sequestration. On the other hand, these particular ecological systems could be considered surrogates for areas of high endemism. The systems are advantageously close together in the Yungas region, are relatively limited in extent (totalling 7% of the study area), and have individual ranges that are < 35% protected.

A final challenge is continued climate change. We know that because of climate change, the ranges of many species will shift across the landscape and possibly out of protected areas [96,97]. Evidence is accumulating that along the Andean slope, species shifts are already occurring [98,99]. Yet the variation in projections of future South American climate makes assessment of the effects on species' distributions difficult [100]. The steep elevation (and therefore climate) gradients in the Andes, where most endemic species are located, suggest that such displacements may take place over relatively small distances. Extinctions are most likely in species inhabiting the highest-elevation habitats, which occur above our study area [100]. Nevertheless, planners should consider adding upslope buffers to conservation areas designated using current distributions of endemic species, and future research could model these species distributions under future climate scenarios.

To complement the further creation and effective management of protected areas, other alternative approaches, which will result in the maintenance of key ecosystems, should expand and continue. These approaches include, strategic conservation on private lands and brokering conservation agreements with private companies, effective land use planning and possibly carbon accounting at the regional government level for both public and private lands, and payments for ecosystem services (e.g. water provision, ecotourism recreation, carbon storage through forests: Reducing Emissions from Deforestation and forest Degradation, REDD). However priority areas for ecosystem services concessions may not necessarily overlap with priorities for biodiversity conservation (e.g.[101]).

## Conclusions

We believe these spatial datasets provide a substantive base upon which to make decisions and move forward for further protection. The approach to developing these datasets described here, relying on existing environmental data sources, data in natural history collections, and in-country expertise to identify endemic species distributions, concentrations and gaps in protection across national borders is applicable to many regions of the world where survey efforts are incomplete. Our results demonstrate that even under these conditions, conservationists can develop spatial datasets for multiple taxonomic groups at a scale useful to guide planning.

## Authors' contributions

Author contributions: All co-authors contributed to data collection and/or analysis of project results. JJS and BEY wrote the majority of the paper with contributions from the co-authors. All authors have read and approved the final manuscript.

## Acknowledgements

We are deeply indebted to the Gordon and Betty Moore Foundation for financial support and technical guidance during the project. The project would not be possible without the generous data sharing, opinions, advice and participation of our long list of collaborators, found at http://www.natureserve.org/aboutUs/latinamerica/col_institutions.jsp. Funds from NatureServe, Duke University's Nicholas School of Environment, and USAID's Emerging Pandemic Threats PREDICT Program supported the publication of this manuscript. The datasets for most of the maps and analyses described here are accessible in both graphic and GIS format at http://www.natureserve.org/andesamazon.

We greatly appreciate the critiques of the anonymous reviewers and Nigel Pitman that helped shape and improve this manuscript.

## Supplementary Material

Additional file 1 **Species distribution modeling, Ecological System mapping, endemism and irreplaceability, gap analysis**. Click here for file

Additional file 2 **Endemic species model results**. Click here for file

Additional file 3 **Gap analysis results**. Click here for file

Additional file 4 **Ecological system accuracy assessment**. Click here for file

Additional file 5 **Sources of species locality data and expert reviewer list**. Click here for file

Additional file 6 **Enlarged map of SE Peru; Vertebrate Endemism & Ecological Systems**. Click here for file

## References

[B1] Myers N Mittermeier RA Mittermeie CG da Fonseca GAB Kent J Biodiversity hotspots for conservation priorities Nature 2000 403 853 858 10.1038/35002501 10706275

[B2] Rodrigues ASL Akcakaya HR Andelman SJ Global gap analysis: Priority regions for expanding the global protected-area network BioScience 2004 54 1092 1100 10.1641/0006-3568(2004)054[1092:GGAPRF]2.0.CO;2

[B3] Orme CDL Davies RG Burgess M Global hotspots of species richness are not congruent with endemism or threat Nature 2005 436 1016 1019 10.1038/nature03850 16107848

[B4] Brooks TM Mittermeier RA da Fonseca GAB Gerlach J Hoffmann M Lamoreux JF Mittermeier CG Pilgrim JD Rodrigues ASL Global biodiversity conservation priorities Science 2006 31 58 61 10.1126/science.1127609 16825561

[B5] Ceballos G Ehrlich PR Global mammal distributions, biodiversity hotspots, and conservation Proceedings of the National Academy of Sciences 2006 103 19374 19379 10.1073/pnas.0609334103 PMC1698439 17164331

[B6] Olson DM Dinerstein E The global 200: priority ecoregions for global conservation Annals of the Missouri Botanical Garden 2002 89 199 224 10.2307/3298564

[B7] IUCN Guidelines for protected areas management categories Cambridge, UK and Gland, Switzerland: International Union for Conservation of Nature 1994

[B8] Patterson BD Ceballos G Sechrest W Tognelli MF Brooks T Luna L Ortega P Salazar I Young BE NatureServe Digital distribution maps of the mammals of the Western Hemisphere 2007 2.0 Arlington, V.A.: NatureServe

[B9] Ridgely RS Allnutt TF Brooks T McNicol DK Mehlman DW Young BE Zook JR NatureServe Digital distribution maps of the birds of the Western Hemisphere 2007 2.1 Arlington, VA

[B10] Schipper J Chanson JS False Chiozza The status of the world's land and marine mammals: diversity, threat, and knowledge Science 2008 322 225 230 10.1126/science.1165115 18845749

[B11] Londoño-Murcia MC Tellez-Valdés O Sánchez-Cordero V Environmental heterogeneity of World Wildlife Fund for Nature ecoregions and implications for conservation in Neotropical biodiversity hotspots Environmental Conservation 2010 37 2 116 127 10.1017/S0376892910000391

[B12] Freitag S Nicholls AO Van Jaarsveld AS Nature reserve selection in the Transvaal, South Africa: what data should we be using? Biodiversity and Conservation 1996 5 685 698 10.1007/BF00051781

[B13] Hurlbert AH White EP Disparity between range map- and survey-based analyses of species richness: patterns, processes and implications Ecology Letters 2005 8 319 327 10.1111/j.1461-0248.2005.00726.x

[B14] Ferrier S Mapping spatial pattern in biodiversity for regional conservation planning: where to from here? Systematic Biology 2002 51 331 363 10.1080/10635150252899806 12028736

[B15] Leroux SJ Schmiegelow FKA Biodiversity concordance and the importance of endemism Conservation Biology 2007 21 266 268 10.1111/j.1523-1739.2006.00628.x 17298533

[B16] Balmford A Mace GM Ginsberg JR G.M Mace AB, and J.R. Ginsberg The challenges to conservation in a changing world: putting processes on the map Conservation in a changing world 1998 Cambridge: Cambridge Univ Press 1 28

[B17] van der Werff H Consiglio T Distribution and conservation significance of endemic species of flowering plants in Peru Biodiversity and Conservation 2004 13 1699 1713

[B18] Roy MS Silva JMCD Arctander P Garcia-Moreno J Fjeldså J Mindell DP The speciation of South American and African birds in montane regions Avian Molecular Evolution and Systematics 1997 New York: Academic Press 325 343

[B19] Fjeldså J Geographical patterns for relict and young species of birds in Africa and South America and implications for conservation priorities Biodiversity and Conservation 1994 3 207 226 10.1007/BF00055939

[B20] Roy MS Torres-Mura JC Hertel F Molecular phylogeny and evolutionary history of the tit-tyrants (Aves: Tyrannidae) Molecular Phylogenetic Evolution 1999 11 67 76 10.1006/mpev.1998.0563 10082611

[B21] García-Moreno J Fjeldså J Chronology and mode of speciation in the Andean avifauna Bonner Zoological Monographs 2000 46 25 46

[B22] Dingle C Lovette IJ Canaday C Smith T Elevational zonation and the phylogenetic relationships of the Henicorhina wood-wrens Auk 2006 123 119 134 10.1642/0004-8038(2006)123[0119:EZATPR]2.0.CO;2

[B23] Patton JL Smith MF MtDNA phylogeny of Andean mice: a test of diversification across ecological gradients Evolution 1992 46 174 183 10.2307/2409812 28564953

[B24] Young KR Churchill; SP, Balslev; H, Forero; E, Luteyn JL. New York Biogeographical paradigms useful for the study of tropical montane forests and their biota Biodiversity and conservation of neotropical montane forests 1995 The New York Botanical Garden

[B25] Young KR Ulloa C Luteyn JL Knapp S Plant evolution and endemism in Andean South America: an introduction Botanical Review 2002 68 4 21 10.1663/0006-8101(2002)068[0004:PEAEIA]2.0.CO;2

[B26] Hughes C Eastwood R Island radiation on a continental scale: Exceptional rates of plant diversification after uplift of the Andes Proceedings of the National Academy of Sciences 2006 103 10334 10339 10.1073/pnas.0601928103 PMC1502458 16801546

[B27] Donoghue MJ A phylogenetic perspective on the distribution of plant diversity Proceedings of the National Academy of Sciences 2008 105 11549 11555 10.1073/pnas.0801962105 PMC2556411 18695216

[B28] Lynch JD Duellman WE The Eleutherodactylus of the Amazonian slopes of the Ecuadorian Andes (Anura: Leptdactylidae) Miscellaneous Publication of the University of Kansas Natural History Museum 1980 69 1 86

[B29] Graham CH Ron SR Santos JC Schneider CJ Moritz C Integrating phylogenetics and environmental niche models to explore speciation mechanisms in Dendrobatid frogs Evolution 2004 58 1781 1793 15446430 10.1111/j.0014-3820.2004.tb00461.x

[B30] Lynch JD Vuilleumier F, Monasterio M Origins of the high Andean herpetological fauna. Págs High Altitude Tropical Biogeography 1986 Oxford: Oxford University Press 478 499

[B31] Santos JC Coloma LA Summers K Caldwell JP Ree R Cannatella DC Amazonian amphibian diversity is primarily derived from late Miocene Andean lineages PLOS Biol 2009 7 0448 0461 10.1371/journal.pbio.1000056 PMC2653552 19278298

[B32] Killeen TJ Douglas M Consiglio T Jørgensen PM Mejia J Dry spots and wet spots in the Andean hotspot Journal of Biogeography 2007 34 8 1357 1373 10.1111/j.1365-2699.2006.01682.x

[B33] Hoorn C Wesselingh FP ter Steege H Bermudez MA Mora A Amazonia through time: Andean uplift, climate change, landscape evolution, and biodiversity Science 2010 330 927 931 10.1126/science.1194585 21071659

[B34] Bush M Lovejoy T Amazonian conservation: pushing the limits of biogeographical knowledge Journal of Biogeography 2007 34 1291 1293 10.1111/j.1365-2699.2007.01758.x

[B35] Rodriguez LO Young KR Biological diversity of Peru: determining priority areas for conservation Ambio 2000 29 329 337

[B36] Fjeldså J Alvarez MD Lazcano JM Leon B Illicit crops and armed conflict as constraints on biodiversity conservation in the Andes region Ambio 2005 34 205 211 16042278

[B37] Fjeldså J Lambin E Mertens B Correlation between endemism and local ecoclimatic stability documented by comparing Andean bird distributions and remotely sensed land surface data Ecography 1999 22 63 78 10.1111/j.1600-0587.1999.tb00455.x

[B38] Hellmayr CE Ueber neue und selteneVögel aus Südperu Verh Ornithol Ges Bayern 1912 11 159 163

[B39] Müller P The dispersal centers of terrestrial vertebrates in the Neotropical realm Biogeographica 1973 2 1 244

[B40] Stattersfield AJ Crosby MJ Long AJ Wege DC Endemic bird areas of the world 1998 Cambridge, UK.: BirdLife International

[B41] Pacheco V Quintana HL Hernandez PA Paniagua L Vargas J Young BE Young BE. Arlington, Virginia: NatureServe Mammals Endemic species distributions on the east slope of the Andes in Peru and Bolivia 2007 40 45

[B42] Pacheco V Ceballos; G, Simonetti J. Mexico City, Mexico Mamíferos del Perú Diversidad y conservación de los mamíferos neotropicales 2002 CONABIO-UNAM 586

[B43] Doan TM Arizábal W Microgeographical variation in species composition of the herpetofaunal communities of Tambopata region, Peru Biotropica 2002 34 101 117

[B44] Duellman WE Duellman WE. Baltimore, MD Distribution patterns of amphibians in South America Patterns of distribution of amphibians 1999 Johns Hopkins Univ Press 255 328

[B45] Reichle S Distribution and conservation status of Bolivian Amphibians 2007 Germany: Rheinische Friedrich Wilhelms Universitaet

[B46] Kessler M The elevational gradient of Andean plant endemism: varying influences of taxon-specific traits and topography at different taxonomic levels Journal Biogeography 2002 29 1159 1166 10.1046/j.1365-2699.2002.00773.x

[B47] Knapp S Assessing patterns of plant endemism in Neotropical uplands Botanical Review 2002 68 22 37 10.1663/0006-8101(2002)068[0022:APOPEI]2.0.CO;2

[B48] León B Young KR Camus; JM, Gibby; M, Johns RJ. Kew, U.K. Distribution of pteridophyte diversity and endemism in Peru Pteridology in perspective 1996 Royal Botanic Garden 77 91

[B49] Luteyn JL Diversity, adaptation, and endemism in Neotropical Ericaceae: biogeographical patterns in the Vaccinieae Botanical Review 2002 68 55 87 10.1663/0006-8101(2002)068[0055:DAAEIN]2.0.CO;2

[B50] Guisan A Thuiller W Predicting species distribution: offering more than simple habitat models Ecology Letters 2005 8 993 1009 10.1111/j.1461-0248.2005.00792.x 34517687

[B51] Guisan A Zimmermann NE Predictive habitat distribution models in ecology Ecological Modelling 2000 135 147 186 10.1016/S0304-3800(00)00354-9

[B52] Kremen C Cameron A Moilanen A Phillips S Beentje H Dransfeld J Fisher BL Glaw F Hijmans R Lees D Aligning conservation priorities across taxa in Madagascar with high-resolution planning tools Science 2008 320 222 226 10.1126/science.1155193 18403708

[B53] Prendergast JR Quinn RM Lawton JH Eversham BC Gibbons DW Rare species, the coincidence of diversity hotspots and conservation strategies Nature 1993 365 335 337 10.1038/365335a0

[B54] Ferrier S Pressey RL Barrett TW A new predictor of the irreplaceability of areas for achieving a conservation goal, its application to real-world planning, and a research agenda for further refinement Biological Conservation 2000 93 303 325 10.1016/S0006-3207(99)00149-4

[B55] IUCN, Conservation International, NatureServe Global amphibian assessment 2006 Version 1.1 IUCN (International Union for Conservation of Nature, Conservation International, NatureServe

[B56] Stephens L Traylor ML University H. Boston, MA Ornithological gazetteer of Peru Museum of Comparative Zoology 1983

[B57] Thomas O New mammals from Peru and Bolivia, with a list of those recorded from the Inambari River, upper Madre de Dios Ann Mag Nat Hist 1901 7 5 148 153

[B58] MaNIS/HerpNet/ORNIS Mammal Networked Information System: Georeferencing Guidelines 2001

[B59] Hijmans RJ Cameron SE Parra JL Jones PG Jarvis A Very high resolution interpolated climate surfaces for global land areas International Journal of Climatology 2005 25 1965 1978 10.1002/joc.1276

[B60] Farr TG The Shuttle Radar Topography Mission Rev Geophys 2007 45 RG2004

[B61] Hansen M DeFries R Townshend JR Carroll M Dimiceli C Sohlberg R Univ Maryland CP. College Park, MD 2003 Vegetation Continuous Fields, MOD44B, 2001 Percent Tree Cover, Collection 3

[B62] Graham CH Ferrier S Huettman F Moritz C Peterson AT New developments in museum-based informatics and applications in biodiversity analysis Trends in Ecology and Evolution 2004 19 497 503 10.1016/j.tree.2004.07.006 16701313

[B63] Loiselle BA Howell CA Graham CH Goerck JM Brooks T Smith KG Williams PH Avoiding pitfalls of using species distribution models in conservation planning Conservation Biology 2003 17 1591 1600 10.1111/j.1523-1739.2003.00233.x

[B64] Nelson BW Ferreira C da Silva M Kawasaki ML Endemism centres, refugia and botanical collection density in Brazilian Amazonia Nature 1990 345 714 716 10.1038/345714a0

[B65] Elith J Graham CH Anderson RP Novel methods improve prediction of species' distributions from occurrence data Ecography 2006 29 129 151 10.1111/j.2006.0906-7590.04596.x

[B66] Phillips SJ Anderson RP Schapire RE Maximum entropy modeling of species geographic distributions Ecological Modelling 2006 190 231 259 10.1016/j.ecolmodel.2005.03.026

[B67] Hernandez PA Franke I Herzog SK Pacheco V Paniagua L Quintana HL Soto HA Swensen JJ Tovar C Valqui TH Predicting species distributions in poorly-studied landscapes Biodiversity and Conservation 2008 17 1353 1366 10.1007/s10531-007-9314-z

[B68] Hernandez PA Graham CH Master LL Albert DL The effect of sample size and species characteristics on performance of different species distribution modeling methods Ecography 2006 29 773 785 10.1111/j.0906-7590.2006.04700.x

[B69] Wisz MS Hijmans RJ Li J Peterson AT Graham CH Guisan A Predicting Species Distributions Working Group N: Effects of sample size on the performance of species distribution models Diversity and Distributions 2008 14 763 773 10.1111/j.1472-4642.2008.00482.x

[B70] Loiselle BA Jørgensen PM Consiglio T Jiménez I Blake JG Lohmann LG Montiel OM Predicting species distributions from herbarium collections: does climate bias in collection sampling influence model outcomes? Journal of Biogeography 2008 35 105 116

[B71] Pressey RL Watts M Ridges M Barrett T C-Plan conservation planning software User Manual 2005 New South Wales, Australia: New South Wales Department of Environment and Conservation

[B72] Josse C Navarro G Comer P Evans R Faber-Langendoen D Fellows M Kittel G Menard S Pyne M Reid M Ecological systems of Latin America and the Caribbean: a working classification of terrestrial systems 2003 Arlington, VA: NatureServe

[B73] Comer P Faber-Langendoen D Evans R Ecological systems of the United States: a working classification of U.S. terrestrial systems 2003 Arlington, VA: NatureServe

[B74] Comer P Schulz K Standardized ecological classification for meso-scale mapping in southwest United States Rangeland Ecology Management 2007 60 324 335 10.2111/1551-5028(2007)60[324:SECFMM]2.0.CO;2

[B75] Sayre R Bow J Josse C Sotomayor L Touval J Campbell JC, Jones KB, Smith JH Terrestrial ecosystems of South America North America land cover summit: a special issue of the Association of American Geographers 2008 Washington, DC 131 152

[B76] Lowry J Ramsey RD Thomas K Mapping moderate-scale land-cover over very large geographic areas within a collaborative framework: a case study of the Southwest Regional Gap Analysis Project (SWReGAP) Remote Sensing of Environment 2007 108 108 59 73

[B77] Scott MJ Davis F Cusuti B Noss R Butterfield B Groves C Anderson H Caicco S D'Erchia F Edwards TC GAP analysis: a geographic approach to protection of biological diversity Wildlife Monographs 1993 123 1 41

[B78] IUCN, UNEP-WCMC The World Database on Protected Areas (WDPA) 2010 Cambridge, UK: UNEP-WCMC

[B79] Calle Josse L Ecological Systems of the Amazon Basin of Peru and Bolivia: Classification and Mapping 2007 Arlington, Virginia: NatureServe

[B80] Ellenberg H Vegetationsstufen in perhumiden bis perariden Bereichen der tropischen Anden Phytocoenologia 1975 2 368 387

[B81] Sieck M Ibisch P Moloney K Jeltsch F Current models broadly neglect specific needs of biodiversity conservation in protected areas under climate change BMC Ecology 2011 11 1 12 10.1186/1472-6785-11-12 21539736 PMC3108268

[B82] Olson DM Dinerstein E Wikramanayake ED Burgess ND Powell GVN Underwood EC D'Amico JA Itoua I Strand HE Morrison JC Terrestrial Ecoregions of the World: A New Map of Life on Earth Bioscience 2001 51 11 933 938 10.1641/0006-3568(2001)051[0933:TEOTWA]2.0.CO;2

[B83] Graves GR Linearity of geographic range and its possible effect on the population structure of Andean birds Auk 1988 105 47 52

[B84] Young BE Franke I Hernandez PA Herzog SK Paniagua L Soto A Tovar C Valqui T Using spatial models to predict areas of endemism and gaps in the protection of Andean slope birds Auk 2009 126 554 565 10.1525/auk.2009.08155

[B85] Young BE Endemic species distributions on the east slope of the Andes in Peru and Bolivia 2007 Arlington, VA: NatureServe

[B86] Phillips SJ Dudík M Elith J Graham CH Lehmann A Leathwick J Ferrier S Sample selection bias and presence-only distribution models: implications for background and pseudo-absence data Ecological Applications 2009 19 1 181 197 10.1890/07-2153.1 19323182

[B87] Leon B Pitman N Roque J Introducción a las plantas endémicas del Perú Rev Peru Biol 2006 13 2 9 25

[B88] Delgado C Is the Interoceanic Highway exporting deforestation? Master's thesis 2008 Durham: Duke University22291979

[B89] Finer M Jenkins CN Pimm SL Keane B Ross C Oil and Gas Projects in the Western Amazon: Threats to Wilderness, Biodiversity, and Indigenous Peoples PLoS ONE 2008 3 8 e2932 10.1371/journal.pone.0002932 18716679 PMC2518521

[B90] Fraser B Peruvian gold rush threatens health and the environment Environmental Science and Technology 2009 43 7162 7164 10.1021/es902347z 19848116

[B91] Swenson JJ Carter CE Delgado CI Domec JC Gold mining in the Peruvian Amazon: global prices, deforestation, and mercury imports PLoS One 2010 6 4 e18875 10.1371/journal.pone.0018875 PMC3079740 21526143

[B92] Killeen TJ Calderon V Soriana L Quezeda B Steininger MK Harper G Solorzano LA Tucker TJ Thirty years of land-cover change in Bolivia: exponential growth and no end in sight Ambio 2007 36 600 606 10.1579/0044-7447(2007)36[600:TYOLCI]2.0.CO;2 18074899

[B93] Margules CR Pressey RL Systematic conservation planning Nature 2000 405 243 253 10.1038/35012251 10821285

[B94] Ferroukhi L Municipal forest management in Latin America 2003 Bogor, Indonesia.: CIFOR and IDRC

[B95] Groves CR Jensen DB Valutis LL Redford KH Shaffer ML Scott JM Baumgartner JV Higgins JV Beck MW Anderson MG Planning for biodiversity conservation: putting conservation science into practice Bioscience 2002 52 499 512 10.1641/0006-3568(2002)052[0499:PFBCPC]2.0.CO;2

[B96] Parmesan C Ecological and evolutionary responses to recent climate change Annual Review Ecology Systematics 2006 37 637 669 10.1146/annurev.ecolsys.37.091305.110100

[B97] Williams P Hannah L Andelman S Midgley G Araújo M Hughes G Manne L Martinez-Meyer E Pearson R Planning for climate change: identifying minimum dispersal corridors for the Cape Proteaceae Conservation Biology 2005 19 1063 1074 10.1111/j.1523-1739.2005.00080.x

[B98] Feeley KJ Silman MR Bush MB Farfan W Cabrera KG Malhi Y Meir P Revilla NS Quisiyupanqui MNR Saatchi S Upslope migration of Andean trees Journal of Biogeography 2011 38 4 783 791 10.1111/j.1365-2699.2010.02444.x

[B99] Forero-Medina G Terborgh J Socolar SJ Pimm SL Elevational Ranges of Birds on a Tropical Montane Gradient Lag Behind Warming Temperatures PLoSOne 2011 6 12 e28535 10.1371/journal.pone.0028535 PMC3233588 22163309

[B100] Magrin G Gay García C Cruz Choque D Giménez JC Moreno AR Nagy GJ Nobre C Villamizar A Parry; ML, Canziani; OF, Palutikof; JP, Linden; PJvd, Hanson CE. Cambridge, U.K Latin America. Climate change 2007: impacts, adaptation and vulnerability Climate change 2007: impacts, adaptation and vulnerability 2007 Cambridge Univ Press 581 615

[B101] Chan KMA Shaw MR Cameron DR Underwood EC Daily GC Conservation Planning for Ecosystem Services PLOS Biol 2006 4 11 e379. 17076586 10.1371/journal.pbio.0040379 PMC1629036

