# Supplementary material for: Plant and animal endemism in the eastern Andean slope: challenges to conservation
Source: BMC Ecol. 2012 Jan 27;12:1. doi: 10.1186/1472-6785-12-1 (PMC3311091; doi:10.1186/1472-6785-12-1)
Supplement: Additional file 1 — Species distribution modeling, Ecological System mapping, endemism and irreplaceability, gap analysis. [file 1472-6785-12-1-S1.DOC]

File 1: Supplemental description of methods

**Part 1. SPECIES DISTRIBUTION MODELING METHODS**

**Overview for all species**

*Adapted from:*

Young, B.E. 2007. Study area. Pp. 8-12 in B. E. Young (editor), *Endemic species distributions on the east slope of the Andes in Peru and Bolivia*. NatureServe, Arlington, Virginia, USA

and

Hernandez, P.A. 2007. Distribution modeling methods. Pp. 13-17 in B.E. Young (editor), *Endemic species distributions on the east slope of the Andes in Peru and Bolivia*. NatureServe, Arlington, Virginia, USA

**A. Selection of species**

We defined the focal species for this study as those that are endemic to our study area. Because ecological boundaries are rarely as sharp as depicted on an ecoregion map, we maintained some flexibility in our criteria for inclusion. As an initial requirement for inclusion in our study, we chose all species with at least 90% of their known range in the study area that had been formally described by 2006. We then identified all species with ranges entirely within the analysis extent which included a buffer of 100 km around the study area. From the resulting list of species, we eliminated all species whose entire range was restricted to the buffer area and, therefore, did not occur in the study area *sensu stricto*. For the species occurring in both the buffer area and the study area, we eliminated all that were restricted to habitat types such as *puna* that did not occur in substantial amounts within the study area. Additionally, for species in humid forests on the northern and eastern boundaries of the study area, we eliminated those for which most known localities lie outside the study area.

In practice, for the three vertebrate groups we developed a Geographical Information System (GIS) algorithm to select species whose distributions met the inclusion criteria. Range maps in GIS format for these groups are available at NatureServe’s website: (http://www.natureserve.org/getData/animalData.jsp). The algorithm compared these maps with the buffered study area to develop a list of candidate species. We refined this list by examining habitat affinities of species in borderline cases and by consulting taxonomic specialists to add recently-described species or to eliminate those with questionable taxonomic status. Selecting endemic plant species was more difficult because of the lack of comprehensive, geospatially explicit distribution data for any of the focal groups (see plant details below). We therefore relied on draft lists of national endemics and input from taxonomic specialists. For some cases, when we were unsure of the distribution of a species, we compiled localities from herbarium records and plotted them on a map of the study area. For species that occurred in both the study area and the buffer zone, we again relied on habitat information to determine whether to include the species.

For plants, we did not include those species for which current taxonomists recognize one or more infraspecific categories (e.g. subspecies, varieties), some of which are reported outside the boundaries of our project’s study areaFor this reason, we did not include species such as *Cavendishia nobilis* (Ericaceae) or *Justicia kuntzei* (Acanthaceae), among others, in our study. We also eliminated species for which the taxonomic status is unclear such that the known localities may refer to more than one biological species (e.g., the mouse opossum *Marmosa quichua*, family Didelphidae). We also had no choice but to eliminate valid species endemic to the study area for which we know of no discrete locality where the species is confirmed present. For example, the hummingbird *Discosura letitiae* (Trochilidae) is known from two localities in Bolivia, but the collections were made well before the era of providing precise location information on specimen tags. Without knowing more details about where this species was found, we cannot predict what its distribution might be. The resulting lists of focal species included 115 birds, 55 mammals, 177 amphibians, and 435 plants.

Because of the tremendous diversity of plants in the region combined with the variable knowledge of families and subgroups, we established the following criteria for inclusion in our study (Young 2007):

1. Taxonomic knowledge. must be well known at the species level in both Peru and Bolivia. We therefore restricted the list to groups with recent monographs describing the characteristics and distribution of each species. The publication of a monograph generally meant that the specimens of the group housed in major herbaria were fairly accurately identified. The knowledge needed to be even for species occurring in both countries to avoid biasing results toward either country. For this reason, for example, we did not treat orchids (Orchidaceae) because although they are well known in Bolivia (e.g., Müller *et al*. 2003), their taxonomy and distribution are less well understood in Peru.

2. General Distribution. Selected groups must show examples of endemism in the study area. For obvious reasons, we did not include families that have few or no species endemic to the study area.

3. Available distribution information. The groups we selected were known to have readily available locality data in herbaria or the literature for use in distribution models. Because of this factor we eliminated groups such as the cacti (Cactaceae) for which many species have been described based on vague locality information. In general, groups treated in recent monographs satisfied this condition.

4. Diversity of life forms. Among the candidate groups, we selected a suite that together represents the range of plant life forms in the study area, including herbs, vines, lianas, shrubs, treelets, and trees. The list includes species that root in the ground as well as those that live as epiphytes or hemiepiphytes.

5. Diversity of elevation. The suite of groups that we selected has endemic species that tend to occur across the range of elevations represented in the study area.

6. Diversity of habitats. The groups we chose also have species with habitat affinities that include all major habitats that occur in the study area. Thus the list includes groups that occur in the mountain forest of the Yungas, lowland moist forests, savannas, and dry valleys.

7. Economic uses. We included groups with species of economic value to help make the results more relevant for the general public and because species with economic uses can become threatened due to overexploitation.

Based on these criteria, we developed a plant list of twelve focal families plus three focal genera from two families to include in the study (Table 1). Besides not addressing obvious candidate families such as the Orchidaceae and Cactaceae as explained above, we did not focus on Pteridophyta other than the Cyatheaceae because a revision of the Bolivian species is not yet complete, Araceae because too many species have yet to be described, Amaryllidaceae because they have received relatively little attention from collectors in our study area, and the Aristolochiaceae because its center of distribution is south of the study area. In summary, the list includes 435 species (complete species list in Appendix S4) ; a variety of lifeforms, with peaks of diversity at different elevational ranges.

Table 1. Descriptive characteristics of the focal groups of endemic vascular plants chosen for species distribution modeling.

| **Group** | **Number of spp endemic to project area^†^** | **Life forms** | **Elevation where diversity peaks** | **Habitats** |
| --- | --- | --- | --- | --- |
| Acanthaceae | 157 | Herbs, shrubs | mid | Savannahs, Yungas, lowland forest |
| Anacardiaceae | 5 | Trees, shrubs | low | Lowland forest, dry valleys |
| Aquifoliaceae | 14 | Trees, shrubs | high | Yungas, lowland forest |
| Bruneliaceae | 10 | Trees | high | Yungas |
| Campanulaceae | 45 | Shrubs, vines | high | Yungas |
| Chrysobalanaceae | 13 | Trees | low | Lowland forest |
| Cyathaceae | 5 | Tree ferns | mid | Yungas |
| Ericaceae | 47 | Shrubs, vines, epiphytes | high | Yungas |
| Fabaceae – *Inga* | 16 | Trees, shrubs | low | Lowland forest |
| Fabaceae- *Mimosa* | 7 | Shrubs | mid | Dry valleys |
| Loasaceae | 19 | Herbs, shrubs | mid, high | Yungas |
| Malpighiaceae | 25 | Trees, vines | mid, high | Dry valleys, Yungas |
| Marcgraviaceae | 7 | Shrubs, hemiepiphytes | mid | Lowland forest |
| Onagraceae – *Fuchsia* | 33 | Shrubs, vines, treelets | high | Yungas |
| Passifloraceae | 32 | Lianas | high | Yungas |

**B. Georeferencing locality data**

Many of the museum and herbarium specimens did not often have geographical coordinates recorded at the time of the sample. Using the recorded description of the sampling location, we used standardized georeferencing methods based on ancillary data. We followed the georeferencing guidelines established by the Mammal Networked Information System (2001), and used gazetteers (e.g. Stephens and Traylor 1983), 1:100,000 topographic maps produced by the Peruvian and Bolivian national cartographic institutes, and 1:250,000 digital databases (Programa Nacional de Informática y Comunicaciones de Naciones Unidas 1998) available for the study area.

**C. Modeling methods**

**Environmental Data**

We used environmental GIS layers describing climatic, topographic and vegetation cover conditions within our study area to develop species distribution models. These environmental data were sourced from four freely available data providers and developed further for our PDM purposes. Each layer was converted to the study’s geographic projection (a customized Lambert Azimuthal Equal Area), resampled to 1-km resolution (if provided at a finer resolution) and clipped to the study area buffered by 100 km, ensuring that geographic coordinates of the pixel boundaries were identical between layers. Even though a number of environmental datasets were available at a finer resolution, a 1-km pixel was selected for distribution modeling because the spatial precision of the species locality data in the majority of cases is low and therefore better matched to environmental data depicted at a coarser (i.e. 1 km) pixel resolution. To remove redundant information, we performed a correlation analysis to identify a subset of climatic variables that were not correlated with each other (correlation coefficient < 0.7) and also not correlated with elevation. This analysis was performed separately for the montane region (>800 m elevation) and the lowland region

The environmental layers obtained and/or derived from four data providers are described below. We were unable to use the Ecological Systems map in the species modeling as it was being created simultaneously. All preparations of these data layers were performed using ESRI ArcInfo Workstation (9.1) unless indicated differently.

*Hole-filled seamless Shuttle Radar Topographic Mission (SRTM) 90 m digital elevation data Version 2.* We derived three topographic layers from the STRM dataset. Data tiles covering the PDM study area were obtained from CGIAR (http://srtm.csi.cgiar.org, version 3 currently available), merged into a single raster layer and resampled to a 1 km pixel resolution. We obtained slope data from this elevation layer by calculating the degree of slope (i.e. maximum rate of change in elevation from each pixel to its 8 neighbors) using the ArcInfo Workstation GRID command SLOPE. The third topographic layer called topographic exposure expresses the relative position of each pixel on a hillslope (e.g. valley bottom, toe slope, slope, and ridge). It is calculated by determining the difference between the mean elevation within a neighborhood of pixels and the center pixel. The difference is determined over a number of neighborhood windows and averaged in a hierarchical fashion (more weight given to the smallest window) to produce a standardized measure of topographic exposure. We calculated topographic exposure using an ArcInfo Workstation application by Zimmerman (2000) on the digital elevation data using three neighborhood windows of 3x3, 6x6 and 9x9.

*Worldclim bioclimatic database (http://www.worldclim.org).*Worldclim provides 19 summary climatic variables of precipitation and temperature for the 1950-2000 time period (Hijmans 2005). It is inadvisable to use all of these variables because colinearity in PDM predictor layers can have adverse effects on model performance. In an effort to identify and remove redundant information in our PDM environmental layer database we performed a correlation analysis to identify a subset of climatic variables that were not correlated with each other and also not correlated with elevation. This analysis was performed separately for the montane region (> 800 m) and lowland region of our study area to derive a list of uncorrelated variables for the two regions for PDM input.

*Moderate Resolution Imaging Spectroradiometer (MODIS) 500m Global Vegetation Continuous Fields (Hansen et al. 2003, http://glcf.umiacs. umd.edu/data/modis/vcf/data.shtml).* We used the percent tree cover layer for South America, in geographic projection.

*MODIS/Terra Vegetation Indices 16-Day L3 Global 1km (NASA EOS data gateway: http:// edcimswww.cr.usgs.gov/pub/imswelcome).*We obtained data tiles covering the study area for the years 2001-2003. We chose the Enhanced Vegetation Index (EVI) instead of the traditional vegetation index NDVI (also available in this dataset) because EVI has proven to be less prone to saturation in high biomass humid forested areas (Huete *et al.* 2002) and therefore more sensitive to canopy variation than NDVI. The EVI data tiles were projected, merged, and exported to geotif images using the MODIS Reprojection Tool (3.2a, available at http://edcdaac.usgs.gov/landdaac/tools/modis/ index.asp) creating a single image for each 16-day time period. These EVI geotif images were entered into a standardized principle components analysis (PCA) utilizing a correlation matrix. We used the remote sensing software ENVI (4.2) for this analysis. PCA is a commonly used data reduction technique of multitemporal remotely sensed imagery (Hirosawa *et al.* 1996).We utilized the first two axes of the PCA for PDM, as they can be interpreted to represent vegetation structure and temporal dynamics respectively. Persistent cloud cover can complicate this sort of analysis, though using images that summarize data for a number of days helps alleviate the problem. Even 16-day periods can be affected by cloud cover, but areas with continuous cloud cover may have similar vegetative characteristics. We created six additional environmental predictor layers by summarizing the three MODIS data layers within moving windows of 2 km or 5 km using the ArcInfo Workstation GRID command FOCALMEAN. A spatial mismatch between the low precision of the species locality data and high precision of the MODIS satellite data may reduce the utility of the MODIS data products for predicting the distribution of our endemic species. Summarizing each MODIS layer within a spatial moving window was an attempt to compensate for this mismatch. Also, summarizing vegetation cover data in this way may be more ecologically relevant because factors influencing habitat selection are not restricted to the site of a species occurrence but also include the conditions of the surrounding landscape (Mazerolle and Villard 1999, Pearce et al. 2001, Johnson et al. 2002).

**Maxent distribution modeling**

The statistical mechanics approach Maxent was an obvious candidate because previous comparative studies demonstrated that it performs well even with small sample sizes (Hernandez *et al*. 2006, Elith *et al.* 2006, Phillips *et al.* 2006). Also the freely available application facilitates modeling many species at one time. To ensure that Maxent was best suited to modeling distributions of Andean species, we compared the success of Maxent and two new promising methods: Mahalanobis Typicalities (a method adopted from remote sensing analyses), and Random Forests (a model averaging approach to classification and regression trees) (resulting in the publication Hernandez et al 2008). We tested each method at predicting ranges of eight bird and eight mammal species using locality and environmental data gathered for our study. We found that Maxent performed very well, producing results that were more consistent across species with widely varying conditions (Hernandez *et al*., 2008). Results of this comparative analysis supported our decision to select Maxent as the inductive species distribution modeling method for our study. Inductive species distribution modeling models were developed using Maxent for all species with two or more unique localities. Maxent is based on a statistical mechanics approach called maximum entropy, meant for making predictions from incomplete information. It estimates the most uniform distribution (*maximum entropy*) across the study area given the constraint that the expected value of each environmental predictor variable under this estimated distribution matches its empirical average (average values for the set of presence-only occurrence data). Detailed descriptions of Maxent’s methods can be found in Phillips *et al*. (2004 and 2006). The algorithm is implemented in a stand-alone, freely available application (http://www.cs.princeton.edu/~schapire/maxent/). Maxent’s predictions are ‘cumulative values’, representing as a percentage the probability value for the current analysis pixel and all other pixels with equal or lower probability values. The pixel with a value of 100 is the most suitable, while pixels closer to 0 are the least suitable within the study area.

We considered only linear and quadratic features because of the low numbers of localities available for our study species and used default settings for Maxent. Maxent automatically chooses all non presence cells as background (assuming there are <10,000). Maxent then computes the distribution is then calculated over the union of background pixels and the presence points.

Four Maxent models were developed for each species using all the available locality data but varying the input environmental layers. MODIS data products have not been extensively used in species distribution modeling to date. Therefore, we created four models for each species to test the utility of incorporating MODIS data products as species distribution modeling predictors and to determine the best way to use these data. Model 1 consisted of either the climatic and topographic variables selected for montane or lowlands regions depending on where the species was primarily distributed. The remaining models included the same climatic and topography layers as model 1. In addition, model 2 included the MODIS layers not summarized, model 3 included MODIS layers summarized within a 2 km moving window, and model 4 included MODIS layers summarized within a 5 km moving window.

**Rule-based distribution modeling and other approaches**

We relied on ‘deductive’ or rule-based species distribution modeling approaches when the species is known from only one locality or when the inductive Maxent approach did not produce a realistic distribution model. Often very little is known about the habitat requirements of these species besides the elevation at which the specimens were collected. For species that occur within regions of high topographic variation, under the specialist’s direction we created presence-absence maps by defining the maximum and minimum elevations at which the species is expected to occur. Then the specialists indicated the areas that should be removed from the predicted distribution as was done for the inductive models. Elevation ranges were often defined by buffering recorded elevations of known localities by 100 to 200 m. For species that occur in regions with low topographic variation (mainly lowland areas) or those for which reliable elevation information was unavailable, the specialists drew a polygon to delineate the predicted distribution. This was achieved mostly by buffering the known localities by one to 10 km (most often 5 km) depending on the dispersal ability of the kind of plant or animal being modeled, or by drawing a polygon to represent the expected distribution region (e.g., riparian areas in a given drainage). For two bird species, we used the ecological systems layer developed for the project as input into a deductive distribution model by either delineating polygons to be included in the prediction or identifying areas to clip out of a model based on elevational range. In cases of very rare or poorly known species, we simply drew a circle around collecting sites with the radius proportional to the taxon-specific potential dispersal distance estimated by the specialists.

1. **Model evaluation**

Expert reviewers determined whether any of the Maxent models produced reasonable results, and if they did, identified the model and threshold that produced the most reasonable map for the species according to our present understanding of its distribution and the habitat available. We did not attempt to partition the data into records used for training the model and those set aside for a statistical model evaluation because of the scarcity and low spatial precision of available locality data. In this situation, review by specialists familiar with the species is often the best way to determine the modeling procedure that produces the most realistic range prediction (Hernandez et al. 2008, Kremen et al. 2008, Loiselle et al. 2008).While we generated model evaluation statistics during our modeling such as the Area Under the Curve (AUC, or ROC, Receiver Operating Characteristics), these were only relied upon as a rough guide because of their artificially high ratings as they rely on randomly generated points.

We sought external review by specialists familiar with the endemic species to produce presence-absence maps of the species. We did this by asking each reviewer to (1) select the best Maxent model generated with the four different ways of incorporating MODIS data, (2) choose a threshold (prediction value above which model predictions are to be considered positive) for the selected model to best represent the distribution of the species, and finally (3) identify predicted areas that should be removed because the species is known not to occur there. This was achieved mostly by drawing a polygon around predicted areas where the experts believed it was likely for the species to occur. We then clipped out all other areas from the predicted distribution. In a few cases we used the elevation layer to remove areas with elevations above or below what would be expected for the species.

During the review of the predictive distribution maps it became evident that Maxent occasionally did not predict occurrence at some localities for which there were records. Further investigation revealed in most cases that these records had errors in geo-referencing or represented misidentifications. Maxent highlighted these probable errors allowing us to correct them and rerun the models with the updated locality data. At times none of the four Maxent models produced a realistic distribution map for the species. For a few species a new environmental variable dataset was hand-selected and resulted in an improved model for that species. In other instances the reviewers felt that Maxent had erroneously excluded from its predictions a valid locality, so we converted the absent prediction to present in an area delineated by the expert reviewers. If all attempts to refine the Maxent model failed to produce an adequate prediction distribution map, we followed a deductive SDM approach to determine the distribution of the species. Draft species models for every species were reviewed by two to seven different reviewers depending on the existence and availability of experts for both Peruvian and Bolivian ranges.

**Method details for species groups**

**A. Birds**

*Adapted from:* Franke, I., Hernandez, P.A., Herzog, S.K., Paniagua, L., Soto, A., Tovar, C., Valqui, T., & B.E. Young g2007.  Pp. 46-53 in B.E. Young (editor), *Endemic species distributions on the east slope of the Andes in Peru and Bolivia*. NatureServe, Arlington,Virginia, USA.

**Compilation of locality records** We requested specimen locality information for the target species from the major local natural history museums as well as all North American museums with significant holdings of specimens from South America (see S8 list of contributors, sources and reviewers). We georeferenced all localities for which the specimen labels did not include geographical coordinates using the sources described in the Vascular Plants section. We supplemented this data set with additional information from the literature by searching the Zoological Record for the target species and their known synonyms. Additionally, we searched for records in the reports of Conservation International’s Rapid Assessment Program (RAP), the Field Museum’s Rapid Biological Inventory (RBI), and the Smithsonian Institution’s Monitoring and Assessment of Biodiversity (SI/ MAB) program. Because most species of birds can be identified by sight or sound in the field by experienced observers, we included observational records compiled by Asociación Armonía (BirdLife International in Bolivia) and largely representing observations made in Bolivia by S. K. Herzog and A. B. Hennessey. During the review process we added additional unpublished records provided by M. Anciães, J. Fjeldså, D. F. Lane, J. P. O’Neil, and T. Valqui. To ensure the accuracy of the coordinates assigned to each locality, two ornithologists with extensive experience in Peru (D. F. Lane and J. P. O’Neil) reviewed maps displaying the localities for each species that occurs in Peru. The staff of Asociación Armonía performed the same function for the Bolivian data.

For more complete coverage, we added records from the literature by searching the *Zoological Record* and other sources for target species and their synonyms. We also included observational and sound-recording records compiled by S. K. Herzog, A. B. Hennessey, J. Fjeldså, D. F. Lane, S. Mayer, J. P. O’Neil, and T.V. To ensure the accuracy of the coordinates assigned to each locality, S.K.H., I.F., D. F. Lane, J. P. O’Neil, and T.V.—all field ornithologists who have extensive experience with these species—reviewed maps displaying the localities for each species. Before running models, we filtered the data to include only unique localities (only one record per species per 1-km^2^ grid cell, our unit of analysis). We eliminated one species, the Coppery Thorntail (*Discosura letitiae*), because both known records are too vague to georeference accurately. In sum, 115 species fulfilled these criteria.

**Model runs and review.** We ran Maxent models using the revised locality data for all species with at least two distinct localities. Three reviewers familiar with the study area and species reviewed the output (see Appendix S4 for results by species). These reviewers determined whether any of the Maxent models produced reasonable results, and if they did, identified the model and threshold that produced the most reasonable map for the species according to our present understanding of its distribution and the habitat available. The reviewers also identified predicted areas of distribution where the species is known not to occur. For most cases in which the Maxent model produced unusable results, we used deductive models based on elevational ranges to depict distributions. In two cases, we reran Maxent with a reduced number of environmental variables to improve the predictions. Only three species, the Scarlet-banded Barbet (*Capito wallacei*), Vilcabamba Tapaculo (*Scytalopus urubambae*), and Sira Tanager (*Tangara phillipsi*), are known from a single locality. We modeled their distribution using the elevational range where the species have been recorded.

**B. Mammals**

*Adapted from:* Pacheco, V., Quintana, H.L., Hernandez, P.A., Paniagua, L., Vargas, J., & B.E. Young. 2007. Mammals.  Pp. 40-45 in B. E. Young (editor), *Endemic species distributions on the east slope of the Andes in Peru and Bolivia*. NatureServe, Arlington, Virginia, USA.

**Compilation of locality records.** We requested specimen locality information for the target species from the major local natural history museums as well as all North American museums with significant holdings of specimens from South America (see Appendix S7, list of contributors, sources and reviewers). We geo-referenced all localities for which the specimen labels did not include geographical coordinates. We supplemented this data set with additional information from the literature by searching the *Zoological Record* for the target species and their synonyms. Larger mammals are rare in collections because of both the time and cost involved in collecting, preparing, and storing specimens and the difficulty in securing collecting permits. We therefore supplemented our sample for large mammals with field observations or reliably identified tracks. These observations are part of the database maintained at the Centro de Datos para la Conservación at the Universidad Nacional Agraria La Molina in Lima, Peru. V. Pacheco and J. Vargas, two mammalogists with extensive field and museum experience in Peru and Bolivia, respectively, reviewed map displays of the locality database to ensure accuracy before running models. In addition, V. Pacheco visited the Colección Boliviana de Fauna in La Paz, Bolivia, to ensure consistency in the identification of small rodents.

**Model runs and review.** We ran Maxent models using the revised locality data for all species with at least two distinct localities. V. Pacheco reviewed the results for Peru and J. Vargas reviewed the results for Bolivia. These reviewers determined whether any of the Maxent models produced reasonable results and if they did, identified the model and threshold that produced the most reasonable map for the species according to our present understanding of its distribution and the habitat available. The reviewers also identified predicted areas of distribution where the species is known not to occur. In cases in which the Maxent model did not produce a useful prediction, we used deductive models. In these cases for montane species with only one record such as *Thomasomys rosalinda*, we used a buffer of 100 m of elevation above and below the known record, reflecting conservatively the likely home range area of the species. For lowland species, we buffered known localities by distances reflecting the probable area over which the particular type of species might disperse. For example, we buffered one locality for the primate *Callicebus ollalae* by 10 km.

**C. Amphibians**

*Adapted from:* Aguilar, C., Arangüena, L. Córdova, J.H., Embert, D., Hernandez, P.A., Paniagua, L., Tovar, C., and B.E. Young. 2007. Amphibians.  Pp. 35-39 in B. E. Young (editor), *Endemic species distributions on the east slope of the Andes in Peru and Bolivia*. NatureServe, Arlington, Virginia, USA.

**Compilation of locality records**. We obtained specimen locality information for the list of 177 target species from the major collections in Peru and Bolivia as well as all North American museums with significant holdings of specimens from South America (see Appendix S7 list of contributors, sources and reviewers). We geo-referenced all localities for which the specimen labels did not include geographical coordinates. We supplemented this data set with additional information from the literature by searching the *Zoological Record* for the target species and their known synonyms. Once we compiled a draft database of locality records, L. Rodríguez and D. Embert, herpetologists with broad knowledge of the amphibians of Peru and Bolivia, respectively, reviewed the data to ensure accuracy and completeness.

**Model runs and reviews.** We ran Maxent models on all species with at least two localities. In addition to variables mentioned in the overview section above, we used, for highland species (those occurring principally above 800 m), a variable that reflected the range of elevations across each 1-km^2^ pixel. A group of nine herpetologists from Peru and Bolivia reviewed the models and suggested criteria for deriving deductive models for both the species known from single localities and those known from multiple localities for which Maxent did not produce a meaningful model (see Appendix S4 for results by species). Criteria used for deductive models included elevation, watershed boundaries, location of land with natural habitat, or a fixed distance related to the dispersal capability of the species involved. In the cases for which Maxent produced useful models, the reviewers selected one of the four models (i.e., using MODIS vegetation data in different manners, see details above in Methods Overview section) that best depicted their understanding of the range, and then identified the most appropriate threshold for developing a predicted presence-absence distribution map.

**D. Plants**

*Adapted from:* Beck, S. G., P. A. Hernandez, P. M. Jørgensen, L. Paniagua, M. E. Timaná, and B. E. Young. 2007. Vascular plants.  Pp. 18-34 in B. E. Young (editor), *Endemic species distributions on the east slope of the Andes in Peru and Bolivia*. NatureServe, Arlington, Virginia, USA.

**Compilation of herbarium records.** We gathered herbarium records for the focal species by systematically searching the collections at major herbaria within the study area and the TROPICOS database of the Missouri Botanical Garden (see Appendix S7. list of contributors, sources and reviewers). We augmented the sample by including records from herbaria where specialists on particular families worked and by searching the literature. Because most specimen labels did not include coordinates for the collecting locations, we geo-referenced the localities using digital gazetteers and maps. To check the accuracy of the geo-referencing, we then sent for review maps of the localities recorded for each species to taxonomic specialists for each group.

**Model runs and review.** After incorporating reviewers’ comments in the data set, we ran Maxent models for each species with the exception of those species known only from single localities. We then convened a group of eight Peruvian and nine Bolivian botanists who were familiar with the species and the study area to review the resulting modeled distributions. With but one exception, these reviewers were different from the taxonomic specialists who reviewed the locality data. All but two of the reviewers did not participate in any other aspect of the study and were therefore unlikely to be biased in their appraisal of the models. For each species, the botanists selected which, if any, of the four Maxent models reflected a realistic depiction of the distribution. In the cases in which a Maxent model was reasonable, the botanists then selected a cut-off threshold to convert the continuous Maxent predictions to presence-absence maps. Again, this selection was based on the botanists’ experience with the species in the field. Finally, the botanists then eliminated from the Maxent prediction areas such as isolated mountain ranges where the species is known not to occur. For cases in which no Maxent model produced a logical representation of a species’ range, the botanists suggested criteria for producing a deductive model. In most cases, the criteria included elevational distributions in the area of the known collections although in others we used the limits of ecological systems where the species occurred. For some wide-ranging species, the Maxent model produced satisfactory results for most but not all of the distribution. For these species, we produced hybrid models using part of the Maxent prediction in one portion of the range and a deductive model for the remaining part. For species known only from single localities, the botanists again selected an elevational range for the species in the vicinity of the locality. In cases of lowland species or those without a known elevational distribution, we simply drew a circle around the locality with a radius appropriate to the dispersal distance of the plant form involved. For example, we typically used a 5 km radius for trees and 2 km for herbs. See Appendix S4 for results by species.

**References for Part I**

Chapman, A.D. and J. Wieczorek. 2006. Guide to best practices for georeferencing. Global Biodiversity Information Facility, Copenhagen.

Elith, J., et al. 2006. Novel methods improve prediction of species’ distributions from occurrence data. Ecography 29:129-151.

Hansen, M., R. DeFries, J. R. Townshend, M. Carroll, C. Dimiceli, and R. Sohlberg. 2003. Vegetation Continuous Fields MOD44B, 2001 Percent Tree Cover, Collection 3. University of Maryland, College Park.

Hernandez, P.A., et al. 2008. Predicting species distributions in poorly-studied landscapes. Biodiversity and Conservation **17**:1353-1366.

Hijmans, R.J., S.E. Cameron, J.L. Parra, P.G. Jones, and A. Jarvis 2005. Very high resolution interpolated climate surfaces for global land areas. International Journal of Climatology 25:1965-1978.

Hirosawa, Y., S. E. Marsh, and D. H. Kliman. 1996. Application of standardized principal component analysis to land-cover characterization using multitemporal AVHRR data. Remote Sensing of Environment **58**:267–281.

Huete, A., K. Didan, T. Miura, E. P. Rodriguez, X. Gao, and L. G. Ferreira. 2002. Overview of the radiometric and biophysical performance of the MODIS vegetation indices. Remote Sensing of Environment **83**:195–213.

Johnson, C. M., L. B. Johnson, C. Richards, and V. Beasley. 2002. Predicting the occurrence of amphibians: An assessment of multiple-scale models. Pages 157–170 in Predicting Species Occurrences: Issues of Accuracy and Scale (J. M. Scott, P. J. Heglund, M. L. Morrison, J. B. Haufler, M. G. Raphael, W. A. Wall, and F. B. Samson, Eds.). Island Press, Washington, D.C.

Kremen, C., et al. 2008. Aligning conservation priorities across taxa in Madagascar with high-resolution planning tools. Science **320**:222-226.

Loiselle, B.A., C.A. Howell, C.H. Graham, J.M. Goerck, T. Brooks, K.G. Smith, and P.H. Williams. 2003. Avoiding pitfalls of using species distribution models in conservation planning. Conservation Biology **17**:1591-1600.

Mammal Networked Information System 2001. MaNIS/HerpNet/ORNIS Georeferencing Guidelines. Available from <http://manisnet.org/GeorefGuide.html> (accessed 2005).

Müller, R., C. Nowicki, W. Barthlott, and P. L. Ibisch. 2003. Biodiversity and endemism mapping as a tool for regional conservation planning – case study of the Pleurothallidinae (Orchidaceae) of the Andean rain forests in Bolivia. Biodiversity and Conservation **12**:2005-2024.

Mazerolle, M. J., and M.-A. Villard. 1999. Patch characteristics and landscape context as predictors of species presence and abundance: A review. Écoscience **6**:117–124.

Pearce, J. L., K. Cherry, M. Drielsma, S. Ferrier, and G. Whish. 2001. Incorporating expert opinion and fine-scale vegetation mapping into statistical models of faunal distribution. Journal of Applied Ecology **38**:412–424.

Phillips, S. J., M. Dudik, and R. E. Schapire. 2004. A maximum entropy approach to species distribution modeling. Proceedings of the Twenty-first Century International Conference on Machine Learning.

Phillips, S.J., R.P. Anderson, and R.E. Schapire. 2006. Maximum entropy modeling of species geographic distributions. Ecological Modelling **190**:231-259.

Stephens, L. and M.L. Traylor 1983. Ornithological gazetteer of Peru. Museum of Comparative Zoology, Harvard University, Cambridge, MA.

Young, B.E. 2007. Endemic species distributions on the east slope of the Andes in Peru and Bolivia. NatureServe, Arlington, Virginia. Available from <http://www.natureserve.org/andesamazon> (accessed 2007).

Young, B.E., I. Franke, P.A. Hernandez, S.K. Herzog, L. Paniagua, A. Soto, C. Tovar, and T. Valqui. 2009. Using spatial models to predict areas of endemism and gaps in the protection of Andean slope birds. Auk **126**:554-565.

Zimmermann, N. 2000. Tools for Analyzing, Summarizing, and Mapping of Biophysical Variables. Available at: [www.wsl.ch/staff/niklaus.zimmermann/progs.html](http://www.wsl.ch/staff/niklaus.zimmermann/progs.html) (accessed 2005).

**Part II. ECOLOGICAL SYSTEM METHODS DESCRIPTION**

**Ecological system classification system**

To identify different ecological types across the Amazon basin of Peru and Bolivia, we applied a recently developed classification system for Latin America and the Caribbean by NatureServe (Josse et al. 2003). Terrestrial ecological systems are defined as groups of vegetation communities that tend to co-occur in the landscape because of their relation with common and determinant factors, such as ecological processes, substrates and/or environmental gradients (Comer et al. 2003). A given terrestrial ecological system will typically manifest itself in a landscape at intermediate geographic scales of dozens to thousands of hectares and persist for 50 or more years. This temporal scale allows the incorporation of the typical succession dynamics to each unit’s concept. With these temporal and spatial scales delimiting the concept of ecological systems, we then integrate multiple ecological factors –or diagnostic parameters- to define each unit. These multiple ecological factors are evaluated and combined in different ways to explain vegetation spatial patterns This system of classification is becoming an emerging standard in Latin America (Sayre et al. 2009, Josse et al. 2009).

This approach was used for the classification of ecological systems of Latin America and the Carribbean (Josse et al. 2003). Considering the great diversity of species in the Neotropics and the relative lack of systematized floristic information, the different phytogeographic classifications existing at floristic region and province scale were used to separate systems that otherwise share the same type of landscape, physical habitat and ecological processes. Another goal of the ecological systems classification is to capture the effect of ecological processes. For example communities constantly alternating between early to midsuccessional stages due to seasonal flooding, constitute a system different to that formed by mature forests that, despite having some degree of seasonal flood have a different disturbance regime due to topography and distance from the river. Finally, the classification of ecological systems is based on the actual and not the potential vegetation coverage. Through a series of expert workshops, diagnostic classifiers were developed to explain the spatial co-occurrence of natural communities; diagnostic classifiers included bioclimate, biogeographic history, physiography, landform, physical and chemical substrates, dynamic processes, landscape juxtaposition, and vegetative structure and composition.

This classification system for Latin America and the Caribbean was carried out with the contribution of numerous vegetation experts, using hundreds of bibliographic and cartographic references that allowed for the identification and description of 758 classes of ecological systems from Mexico to Tierra del Fuego (Josse et al. 2003). This *a priori* classification has been applied in numerous regional mapping efforts, where the most appropriate means to delimit the identified units were sought. It was also recently modeled and mapped across the continent of South America based on environmental and land cover data and expert classification (Sayre et al. 2008). The classification system is still evolving, with adjustments to the existing unit concepts or the addition of new ecosystem types as they are described and reviewed, usually in the course of mapping applications.

For our study area in Peru and Bolivia, we started with the systems identified by Josse (2003) for the region, and refined them based on an additional series of meetings of vegetation and mapping experts from Peru and Bolivia. The Josse et al. (2003) is an *a priori* classification and enabled a relatively independent process where the most appropriate means to delimit the identified units are sought. Depending on the spatial scale and the ability to distinguish specific types with the mapping methods, this process results in legend adjustments, joining two ecological systems for example that cannot be separated or depending on the complexity of spatial pattern, determining vegetation complexes of different interspersed types. For some systems there are complex mosaics impossible to separate at this scale, such as the Beni savannahs and some Amazon riparian systems.

**Data and methods overview for entire study area**

We chose a minimum mapping unit of 25 hectares, and a working spatial scale of 1:250.000. We based our mapping primarily on field work, satellite imagery of 30 m resolution (Landsat 5 and 7 Thematic Mapper & Enhanced Thematic Mapper +), and other spatial data sources such as elevation and bioclimatic information. Existing maps were used as starting point, yet they were all thoroughly modified to adjust to the vegetation classification requirements and spatial resolution of this project. Many small areas had not been mapped previously, and one large area, the Beni flooded savannah, had never been mapped before this effort. Our study area was stratified into three mapping areas that are geographically distinct and were represented by three different mapping teams with regional familiarity and expertise. The first region was the Peruvian uplands, eastern slope or ‘Yungas” that covers varied Andean topography from elevations of 800 to 3600 m. The mapping of this area was completed by the Centro de Datos para la Conservacion (CDC) Universidad La Molina- Lima, Peru, in collaboration with NatureServe staff. The Peruvian lowlands, areas < 800 m, were mapped by the Instituto de Investigaciones le la Amazonia Peruana (IIAP), Iquitos, Peru in collaboration with NatureServe, and the Bolivian areas were mapped by a team of independent mapping experts Rumbol SRL (Navarro, G. & W. Ferreira). These mapping teams were identified after broad in-country consultations.

The mapping approaches of the three teams were similar and were all based on the same satellite imagery as well as integrating of field samples and ancillary data. The ancillary data used in mapping included bioclimate (Worldclim and others), geology, geomorphology, and any existing information on vegetation. Generally the teams spatially stratified their extensive regions by the ancillary data depending on the type of terrain and vegetative patterns. Within the stratified subregions, detailed mapping of ecological systems was done by teams of experts familiar with the terrain and vegetation and was either conducted by spatial GIS modeling and image classification (Peruvian uplands), or by on-screen digitizing and image classification for some areas.

We had initially hoped to implement more advanced image classification methods (e.g. Classification and regression trees (CART) used for US southwestern mapping in Lowry et al. 2006), yet were limited by, in order of importance, a lack of ground truth points (the former reference had ~93,000 field points for a similar sized area), a lack of familiarity with these advanced classification methods for some of our mapping teams, as well as a limited 2.5 year project timeline. That said we are not entirely certain that a more advanced method such as CART would be appropriate for our extremely varied and large study area, given that the field data it would require would be substantial. The satellite image interpretation relied heavily on ecosystem familiarity of the well-traveled mapping teams; such integrative knowledge would be difficult to include in these more advanced methods.

**A. Satellite imagery**

Imagery covering the study area consisted of ~100 Landsat Thematic Mapper images. Images for Bolivia and the Andean region of Peru were dated from the late 1990s and more recent, and areas of recent land use change in the Peruvian Amazon date back to 2003 to 2005. Only a few of the most inaccessible areas of the Peruvian Amazon are represented by images from 1980’s because of continuous cloud cover and have not had notable human intervention. Landsat imagery at the time cost ~US$400 an image to purchase. Much of the imagery had been previously acquired by the local organizations for previous mapping efforts and a selection if images were purchased to update areas in Peru. Many of the images were part of the NASA Global Mosaics dataset available free of charge at the time of the project (Tucker et al. 2004). All images were georectified based on GPS points as well as 1:100000 National Planimetric Map. Imagery for the lowlands for Peru and Bolivia was radiometrically scaled as mosaics used for visual interpretation, relying on visible and near infrared bands.

**B. Field sampling**

Field data was collected during the dry seasons of 2005 and 2006. A number of field samples were gathered for many areas of the study area with slightly different objectives. Field samples in Bolivia and upland Peru were designed to cover as many different ecological systems as possible for map creation and accuracy assessment, while in addition to these objectives, the field surveys in the Amazonian lowlands of Peru included intensive plant and soil sampling for some lesser known ecological systems. Samples were distributed across the study area as much as our project timeline and accessibility would allow. Most samples were, however located within a few km of a road or river. We also relied on gathering previously sampled inventory points (n=236), to include in our database.

We initially approached the problem of where to sample in the field by using a spatial optimization tool, SPOT (Spatial Optimization Tool, Shoutis 2003), which suggests areas with high representation of ecological systems that were relatively accessible. Thus our approach was a stratified cluster sample. We stratified the area by draft ecological systems (in some cases of previously unmapped areas, we relied upon the continental-wide modeled ecosystem map, Sayre et al. 2008). And then identified clusters of sample points by the optimization routine which was run across the landscape based on the geospatial layers: digital elevation model, draft ecological systems map, populated centers, roads, and navigable rivers. Criteria for the SPOT selection were: 1) to avoid non forest or disturbed 2) include a wide range of elevations 3) include as many [intersecting] ecological systems as possible 4) within 2 hours travel time from a navigable road. Areas were selected that would provide the opportunity to obtain cluster samples for either map creation/validation or accuracy assessment.

A requirement for all field plots was that they be centered in an area of homogenous vegetation covering at least 1 ha. We designed both a rapid field sampling protocol and more detailed sampling method accompanied by two different database forms for the three teams to use. All teams were equipped with digital cameras and GPSs having < 20 m locational error. For the rapid field plots, teams collected photos, location, elevation, interpreted which ecological system covered the plot, the confidence they had in that interpretation, type of landscape (geological formation), and level of human intervention. Detailed plots recorded additional information on indicator species, soil texture, canopy cover etc (Forms can be found in Josse et al. 2007).

Overall, the Peruvian uplands and areas in Bolivia had adequate road networks to access many geographically disparate areas. Vast regions of the Peruvian Amazon lowlands however, remain inaccessible and relatively unknown for lack of transportation corridors presence of illegal crops and tribal areas. To remedy this we commissioned a targeted aerial flight of high resolution digital photography. We found the photos useful in determining coarse compositional differences (e.g. identification of palm), but limited in distinguishing closely related ecological systems. We also ran a series of expeditions up remote rivers for periods of 7-14 days at a time, where field workers lived on the boat and sampled quick plots alongside the river and more detailed plots walking in through the forest. Field expeditions in the Peruvian lowlands were made along the Madre de Dios, Ucayali, Napo, Tambos, Huallaga, Pastaza, Morona, and Tigre rivers (descriptive information on plots and locations can be found in Josse et al. 2007).

While field sampling was an important part of the ecological system mapping, the project budget and timeline only allowed for limited sampling across our 1.25 million km^2^ study area. We gathered many points for the mapping teams to validate and update their maps, while ~ 25% of the points were reserved for accuracy assessment after the map’s completion.

**C. Accuracy assessment**

The goal of this project was to collect a stratified *random* sample, but similar to most ecological and land cover accuracy assessments, significant logistical and financial constraints affected the sample selection process, and in reality the field and image interpreted samples in this project more often represented stratified *cluster* samples. Field samples were often collected in clusters due to the difficulty of moving through dense jungle and limited air photo image coverage. Stratified *random* cluster samples were implemented for the aerial photo transects, in which photos used for interpretation were selected randomly along the flight transect. In an attempt to increase the spatial independence of the samples, a minimum distance of at least 1000 m was maintained between any field or image interpreted sample point. Approximately 25% of our field samples were used for accuracy assessment; the other 75% were used in map development. Our goal for the minimum number of validation points for the largest ecological system was 30, yet unfortunately, for many ecological systems it was not possible to obtain such a number of samples (this goal would mean obtaining 120 field samples for each system). We report accuracy for all points used in accuracy assessment and accuracy for those systems with at least 8 field samples but provide the complete confusion matrix for the different mapping regions in Appendix S6.

Assessment was not done by an independent team, but by the mapping teams themselves which has the potential to introduce bias in the results. Because of the vast landscape covered and the difficulty of reaching many parts of the study area the most practical and cost effective approach was to have the mapping teams simultaneously gather data for map creation and accuracy assessment at the same time. As well, given the project timeline, field expeditions had to be run during the two dry seasons over the project’s timeline, rather than after the final map was complete.

To assess the maps accuracy we used a confusion matrix for a detailed assessment of individual ecological systems. We also employed a more coarse evaluation matrix for five generalized grouping of different systems. We were able to successfully assess the accuracy of 21 ecological systems in Peru and 45 in Bolivia. Results of the accuracy assessments for the three mapping zones can be found in Appendix S6.

**Methods specific to the three mapping zones**

**A. Peruvian uplands**

The Andean region was covered by ecological systems maps developed within the framework of two Ecoregional Conservation Planning’s projects, sponsored by the Nature Conservancy; one for the Ecoregion of the Eastern Cordillera Real Oriental, carried out by the Ecuadorian Foundation of Ecological Studies (EcoCiencia), comprising a relatively small extension of the Peruvian territory north of the Maranon river. The second one corresponds to the map of terrestrial ecological systems of the Peruvian Yungas, executed by the Conservation Data Center (CDC– UNALM). Both maps have been improved within the context of the present project, thanks to field samples taken for validation and adjustment of the bioclimatic map used as a resource.

An initial classification of 23 Landsat TM images was done to separate into vegetative life-forms of forest, shrubland, grassland, and deforested/degraded across the area. The Peruvian uplands were stratified into three general ecological zones. Within each zone the vegetative life-form map was combined with binned (or classified) environmental variables of elevation, landform, and bioclimate. Landform was created based on the topography and a GIS moving window analysis and bioclimate was represented by the Rivas-Martínez index (Rivas-Martinez & Maldonado 1999; Rivas-Martinez 2000) based on temperature and precipitation. These four categorical layers were then overlain into a cartographic model and unique combinations of these input variables were used to label ecological systems. This was an iterative process where the output was reviewed and initial bin ranges and subsequent labels were revised. (see Josse et al. 2007 for a detailed description of environmental variables and their bins ranges). The number of field samples used in accuracy assessment was 241, yet these were widely distributed across many different ecological systems. In the end, only the four largest ecological systems could be adequately sampled for the individual accuracy assessment. Using a generalized grouping of systems (based on ecological similarity), five general groups of many ecological systems were evaluated for accuracy for a more general characterization of map quality.

**B. Peruvian lowlands**

Previous to this project, the Instituto de Investigaciones de la Amazonía Peruana (IIAP) had been working on the preparation of a mosaic of Landsat TM images (BIODAMAZ, 2004), which was visually interpreted in terms of the 19 types of natural vegetation for the Peruvian Amazon (BIODAMAZ, 2004b).

The BIODAMAZ preexisting map was created based on a series of intersected (vector) variables: geology, geomorpholgy, bioclimate, hydrography, general vegetation types, and known ecological sub regions. The resulting divisions were field tested using stratified sampling during our study, and based on the similarity of plot inventories of tree genera. These areas were further adjusted to ecological systems types, delimited with visual image interpretation with on screen digitizing integrated and further field knowledge and verification. The images had been radiometrically scaled and matched to allow for the best separation of vegetation types using visible and infrared bands. The number of field samples used in accuracy assessment was 270 while 514 assessment samples were derived from digital high resolution air photo interpretation. Accuracy was evaluated for individual systems as well as for five general groupings of those systems.

**C. Bolivian uplands and lowlands**

A new vegetation map of Bolivia was being developed at the time of our project by Rumbol SRL funded by The Nature Conservancy (Navarro & Ferreira 2007). We enhanced and continued this effort to include additional areas as well as produce a legend based on the ecological systems classification (Josse et al. 2003). Previous to this effort there were some areas of detailed mapping by the same team, which were incorporated as well (Amboró/Madidi Corridor--World Wildlife Fund & CISTEL, Pando Department –WWF & HERENCIA, Amboró National Park, Fundacion Amigos de la Naturalez & Wildlife Conservation Society, and northern Isiboro Secure National Park, Beni –CIDDEBENI.) The ecological systems map for the Beni savannahs in Bolivia was first to cover the region in detail. It was developed during this project and was not previously or concurrently mapped by other efforts.

Navarro, G., and W. Ferreira mapping approach in Bolivia is based on the Vegetation Series concept, where physical landscape units are further characterized in terms of the vegetation communities that are diagnostic of these units including all the successional series of each community type and its diagnostic floristic composition (Navarro & Maldonado 2002; Mapa de vegetación de Bolivia. The Nature Conservancy & Rumbol SRL). The mapping was based on the conceptual approach of bioclimatic zones (*pisos*), vegetation zones and series, and geoseries explained in detail in Navarro & Maldonado (2002). An initial cartographic overlay was conducted using maps of geology, soils, vegetation zones, elevation, biogeography, and bioclimate. The edaphic and geomorphological stratification of the lowlands was based on soils analyses published by different sources. Bioclimate was represented by the Rivas-Martínez index (Rivas-Martinez & Maldonado 1999; Rivas-Martinez 2000) based on temperature and precipitation data from the Bolivian National Meteorological Service. Within these resulting divisions, ecological systems were defined from visual image interpretation, overlaying thousands of field points with on screen digitizing and subsequent field validation and reconnaissance. The majority of interpretation was done in false color (4, 7, 2 RGB), to highlight dense, leafy vegetation in red. Interpretation was conducted on the basis of the vegetation’s reflectance, texture or roughness, spatial arrangement and geographic context.

Three hundred field samples were provided for the accuracy assessment. Thirteen samples were dropped from the assessment: three samples occurred outside of the ecological systems map extent and ten samples were within 1000 meters of another sample. A total of 287 viable samples were used in the accuracy assessment. Many systems were assessed for accuracy individually, while systems were grouped into 12 general categories of which 9 general groups had enough points with which to evaluate accuracy.

**References for Part II**

BIODAMAZ (Proyecto Diversidad Biológica de la Amazonía Peruana). 2004. Technical Document 12, BIODAMAZ-IIAP Series, Instituto de Investigaciones de la Amazonía Peruana, Iquitos, Peru.

CDC-UNALM (Centro de Datos para la Conservación, Universidad National Agraria, La Molina. Unpublished Data. Ecological systems map of the Yungas. Lima, Peru.

Comer, P., et al. 2003. Ecological systems of the United States: a working classification of U.S. terrestrial systems. NatureServe, Arlington, VA.

Josse, C., et al. 2003. Ecological systems of Latin America and the Caribbean: a working classification of terrestrial systems. NatureServe, Arlington, VA. Available from http://www.natureserve.org/library/LACEcologicalSystems.pdf (accessed 2005).

Josse, C., et al. 2007. Ecological systems of the Amazon Basin of Peru and Bolivia: classification and mapping. NatureServe, Arlington, Virginia. Available from http://www.natureserve.org/andesamazon (accessed 2007).

Josse, C., F. Cuesta, G. Navarro, V. Barrena, E. Cabrera, E. Chacón-Moreno, W. Ferreira, N. Peralvo, J. Saito, and A. Tovar. 2009. Ecosistemas de los Andes del Norte y Centro. Bolivia, Colombia, Ecuador, Perú y Venezuela. Secretaría General de la Comunidad Andina, Programa Regional ECOBONA-Intercooperation, CONDESAN-Proyecto Páramo Andino, Programa BioAndes, EcoCiencia, NatureServe, IAvH, LTA-UNALM, ICAE-ULA, CDC-UNALM, RUMBOL SRL. Lima, Peru.

Lowry, J. et al. 2007. Mapping moderate-scale land-cover over very large geographic areas within a collaborative framework: a case study of the Southwest Regional Gap Analysis Project (SWReGAP). Remote Sensing and Environment 108:59-73.

Navarro, G. and M. Maldonado. 2002. Geografía Ecológica de Bolivia. Vegetación y Ambientes Acuáticos. Editorial Centro de Ecología Simón I. Patiño. Cochabamba. 719 p.

Navarro, G., and W. Ferreira. 2007. Mapa de vegetación de Bolivia. The Nature Conservancy & Rumbol SRL. Available from <http://www.fan-bo.org/> (accessed 2007).

Rivas-Martín, S., D. Sánchez-Mata and M. Costa. 1999. North American Boreal and Western Temperate Forest Vegetation. Itinera Geobotanica **12**: 5 – 316.

Rivas-Martínez, S. 2000. Global Bioclimatics (Clasificación Bioclimática de la Tierra). Unpublished draft document. Phytosociological Research Center. Madrid.

Sayre, R., J. Bow, C. Josse, L. Sotomayor, & J. Touval. 2008. Terrestrial ecosystems of South America. Pages 131-152 in Campbell, J.C., K. Bruce Jones, & J.H. Smith (eds.), North America Land Cover Summit: A special issue of the Association of American Geographers.

Shoutis, D. 2003. The Spatial Optimization Tool (SPOT). The Nature Conservancy. Available from <http://www.conserveonline.org/workspaces/spot> (accessed 2005)

**Tucker C.J.,** D.M. Grant, & J.D. Dykstra. 2004. NASA's global orthorectified landsat data set. Photogrammetric Engineering and Remote Sensing **70**: 313-322.

**Part III. IRREPLACEABILITY, AREAS OF ENDEMISM, AND GAP ANALYSIS**

**Irreplaceability**

**A. Methods**

We calculated summed irreplaceability as the likelihood that an analysis pixel must be protected to achieve a specified conservation target for the study area (Pressey et al. 1994, Pressey 1999, Ferrier et al. 2000). Modeling with C-PLAN conservation planning software (Pressey et al. 2005), we used 10-km^2^ analysis pixels and set 25 of these pixels for each species as a conservation target. Although this target is somewhat arbitrary, spatial patterns of irreplaceability are typically robust to target level (Rissler et al. 2006). If a species occurs in <25 of the 10-km^2^ pixels, we set the target as the number of pixels in which the species occurs. For each species, irreplaceability for each pixel ranges from 0 to 1. Low numbers indicate that a species occurs in many pixels, whereas values close to 1 reflect the existence of species with very restricted ranges. Summed irreplaceability sums the irreplaceability values for all species occurring at each pixel, drawing attention to the sites (pixels) with the greatest numbers of narrow-ranging species. Summed irreplaceability incorporates the concept that the species with the smallest ranges offer the fewest options for conservation, just as weighted endemism (the sum of the inverse of each species’ range that overlaps each pixel, also known as “range-size rarity”; Knapp 2002) does, but additionally incorporates the complementarity of sites for protecting suites of species. To summarize the results, we divided pixels into six classes of irreplaceability using natural breaks.

**B. Results**

**Birds**

Summed irreplaceability analysis highlighted 10 areas important for narrow-ranging endemics (representative species given for each): (1) white-sands forests near Iquitos (Ancient Antwren and Allpahuayo Antbird); (2) Rio Morona (White-masked Antbird); (3) Cordillera de Colán and the Alto Mayo region (Long-whiskered Owlet, Marvelous Spatuletail, Speckle-chested Piculet, Ochre-fronted Antpitta, and Johnson’s Tody-Tyrant); (4) southeastern La Libertad Department (Yellowbrowed Toucanet and Golden-backed Mountain-Tanager); (5) Tingo María-Carpish Hills (White-tufted Sunbeam, Bay Antpitta, and Tschudi’s Tapaculo); (6) southwestern Cordillera de Vilcabamba (Vilcabamba Tapaculo, Inca Wren, and Vilcabamba Brush-Finch); (7) Cordillera de Urubamba (White-browed Tit- Spinetail); (8) Valle de Pilcopata (Fine-barred Piculet and Blackbacked Tody-Flycatcher); (9) upper Consata watershed (Yungas Tyrannulet and Green-capped Tanager); and (10) Cordillera Muñecas (Berlepsch’s Canastero). Further discussion of these areas and comparisons of patterns to previous studies can be found in Young et al. 2009.

**Plants**

Because many of the focal groups had small numbers of endemic species, we calculated summed irreplaceability for the entire sample of vascular plants. Species known from single localities were common and thus received heavy weighting for their tiny ranges. The result is a map that shows small peaks of irreplaceability near Iquitos, Peru, and throughout the length of the upper portion of the study area in the Andes (Figure 19 in Young 2007). Peaks at Iquitos reflect the occurrence there of a few species of Chrysobalanaceae restricted to the white-sand forests.

**Amphibians**

The analysis of summed irreplaceability reflects the large numbers of amphibians with very small ranges, especially in the north (see Figure 23 in Young 2007). Isolated peaks occur in many parts of the study area. Several are clustered in northern San Martin and adjacent Amazonas Departments, with additional peaks in Ayacucho, Cusco, and Cochabamba Departments

**Mammals**

Weighting the analysis to highlight areas where the smallest-ranged species occur produced a slightly different result. The narrow band from Cusco to La Paz and in Cochabamba disappears. However, five areas stand out as particularly important: (1)

The La Libertad-San Martin border in the Cordillera Central. Location of the Abiseo River National Park and home to endemic species such as *Thomasomys apeco* and *T. macrotis*. (2)West side of the Apurimac river in Ayacucho east to southeastern Madre de Dios. The area upslope from the river including the localities Huanhuachallo and Santa Rosa river, which are the only places where the bats *Sturnira nana* and *Mimon koepckeae* are known to occur, extending east to just into Madre de Dios. (3) Northern tip of the Cordillera de Vilcabamaba. An isolated ridge in Otishi National Park, Junín Department, Peru, where the microendemic Ashaninka Arboreal Chinchilla Rat (*Cuscomys ashaninka*) and Ashaninka Thomasomys (*Thomasomys onkiro*) were discovered (Emmons 1999, Luna and Pacheco 2002). (4) Western Beni Department, Bolivia. A small area near the border with the department of La Paz where the microendemic Ollala Brothers’ Titi (*Callicebus olallae*) and Río Beni Titi (*Callicebus modestus*) occur. (5) Cordilleras near La Paz, Bolivia. This small area supports many endemics, including several with relatively large ranges and a few more restricted species, such as the arboreal mouse Rhagomys longilingua.

**Areas of endemism**

We layered all overlapping species to create species richness. We then chose a threshold to isolate areas of the highest species richness or density. After thoroughly examining the literature for guidance and not finding recommendations, we subjectively chose a threshold of 2/3 of the overlapping species. Raising this threshold would include fewer species and likely result in a smaller area and lowering it would include more species and more area. Resulting areas of endemism are described in Table 1.

Table 1 . Results Summary of areas of endemism identified in this project, in order from north to south.

| Area | Department | Country | Vascular Plants | Amphi-bians^1^ | Mam-mals^1^ | Birds^1^ | Examples of Endemic Species | Protected Areas^2^ |
| --- | --- | --- | --- | --- | --- | --- | --- | --- |
| Iquitos | Loreto | Peru | Chrysbalanaceae, *Inga*, Malpighiaceae |  |  |  | *Hirtella revillae, Inga gereauana, Heteropterys actinoctenia* | -- |
| Tarapoto | San Martín | Peru | *Inga,* Malpighiaceae |  |  |  | *Inga cynometrifolia, Hiraea christianeae* | -- |
| Cordillera Central in Amazonas and San Martín | Amazonas, San Martín | Peru | Loasaceae, Aquifoliaceae |  |  |  | *Nasa formosissima, Ilex tarapotina* | Cordillera de Colán Reserved Zone, Río Abiseo National Park |
| Carpish Hills | Huánuco | Peru | Acanthaceae, Aquifoliaceae, *Fuchsia* |  |  | X | *Aphelandra mucronata, Ilex aggregate, Fuchsia ceracea, Nephelornis oneilli* | -- |
| Cocha Cashu | Madre de Dios | Peru | *Inga* |  |  |  | *Inga megalobotrys* | Manu National Park |
| Cordillera de Vilcabamba | Cusco | Peru | *Fuchsia* |  | XX | XX | *Fuchsia tunariensis, Mormopterus phrudus, Atlapetes canigenis* | Machu Pichu Historical Sanctuary |
| Paucartambo-Marcapata | Cusco (and marginally in Madre de Dios) | Peru | Aquifoliaceae, *Fuchsia* |  | X | XX | *Ilex crassifolioides, Fuchsia vargasiana, Rhagomys longilingua, Iridosornis reinhardti* | Manu National Park, Megantoni National Sanctuary |
| Northwestern Puno | Puno | Peru |  |  | XX | X | *Delothraupis castaneoventris, Akodon aerosus,* | -- |
| Cordillera de Apolobamba | La Paz | Bolivia | Brunelliaceae, Ericaceae, Marcgraviaceae |  |  | XX | *Brunellia boliviana, Thibaudia axillaries, Sarcopera oxystilis, Thomasomys ladewi, Tangara meyerdeschauenseei* | Madidi National Park, Apolobamba Natural Integrated Management Area |
| Cordilleras near La Paz | La Paz | Bolivia | Acanthaceae, Aquifoliaceae, Brunelliaceae, Campanulaceae, Ericaceae,  Marcgraviaceae, *Mimosa*, Passifloraceae |  | X | X | *Justicia albadenia, Ilex pseudoebenacea, Brunellia coroicoana, Centropogon bangii, Sphyrospermum sessiliflorum, Souroubea stichadenia, Oryzomys levipes, Atlapetes rufinucha* | Cotapata National Park and Integrated Management Area, Madidi National Park |
| Cordillera de Cocapata-Tiraque | Cochabamba | Bolivia | Brunelliaceae, *Fuchsia* |  | X | XX | *Brunellia rhoides, Fuchsia garleppiana, Akodon siberiae, Hemitriccus spodiops* | Tunari National Park |
| Central Cochabamba | Cochabamba | Bolivia |  | XX |  | X | Simoxenops striatus | Carrasco National Park |

^1^ XX = Major area of endemism; X = minor area of endemism

^2^ Only national-level protected areas are listed. Additional local or private reserves may also occur.

**Regional gap analysis of protected areas**

**Methods**

This vast study area encompasses 1.25 million km^2^ and includes 42 distinct protected areas designated, or proposed, for conservation. In this analysis, we did not include areas designated or proposed at sub-national levels, such as those established by regional governments and municipalities. Designated national protected areas have been categorized according to the predominant management regime in accordance with classes established by IUCN (Table 2). These management classes include categories I-VI, ranging from strict reserve (I) (not occurring in our study area), to national park (II), national monument (III), managed habitat (IV), protected landscape (V), and sustainable use areas (VI).

While IUCN management categories indicate much about the intended uses within protected areas, they may indicate little about the actual or projected future conditions within and around protected areas.

Table 2. IUCN category definitions for protected areas used in conservation gap analysis

| **Category** | **Definition** |
| --- | --- |
| I - Strict nature reserve/ wilderness area | **Protected area managed mainly for science or wilderness protection.** These areas possess some outstanding ecosystems, features and/or species of flora and fauna of national scientific importance, or they are representative of particular natural areas. They often contain fragile ecosystems or life forms, areas of important biological or geological diversity, or areas of particular importance to the conservation of genetic resources. Public access is generally not permitted. Natural processes are allowed to take place in the absence of any direct human interference, tourism and recreation. Ecological processes may include natural acts that alter the ecological system or physiographic features, such as naturally occurring fires, natural succession, insect or disease outbreaks, storms, earthquakes and the like, but necessarily excluding man-induced disturbances. |
| II - National park | **Protected area managed mainly for ecosystem protection and recreation.** National parks are relatively large areas, which contain representative samples of major natural regions, features or scenery, where plant and animal species, geomorphological sites, and habitats are of special scientific, educational and recreational interest. The area is managed and developed so as to sustain recreation and educational activities on a controlled basis. The area and visitors' use are managed at a level which maintains the area in a natural or semi-natural state. |
| III - Natural monument | **Protected area managed mainly for conservation of specific natural features.** This category normally contains one or more natural features of outstanding national interest being protected because of their uniqueness or rarity. Size is not of great importance. The areas should be managed to remain relatively free of human disturbance, although they may have recreational and tourist value. |
| IV - Habitat/species management area | **Protected area managed mainly for conservation** **through management intervention.** The areas covered may consist of nesting areas of colonial bird species, marshes or lakes, estuaries, forest or grassland habitats, or fish spawning or seagrass feeding beds for marine animals. The production of harvestable renewable resources may play a secondary role in the management of the area. The area may require habitat manipulation (mowing, sheep or cattle grazing, etc.). |
| V - Protected landscape/seascape | **Protected areas managed mainly for landscape/seascape conservation and recreation.** The diversity of areas falling into this category is very large. They include those whose landscapes possess special aesthetic qualities which are a result of the interaction of man and land or water, traditional practices associated with agriculture, grazing and fishing being dominant; and those that are primarily natural areas, such as coastline, lake or river shores, hilly or mountainous terrains, managed intensively by man for recreation and tourism. |
| VI - Managed resource protection area | **Protected area managed for the sustainable use of natural ecosystems.** Normally covers extensive and relatively isolated and uninhabited areas having difficult access, or regions that are relatively sparsely populated but are under considerable pressure for colonization or greater utilization. |

*Source:* McNeely and Miller 1984.

The map of ecological systems, individual species distribution models, as well as areas of endemism and irreplaceability for all species groups were overlain geographically by the national protected area network to evaluate the degree to which they were included within protected area boundaries. We included all protected areas that correspond to World Conservation Union (IUCN) scores I–VI (IUCN 1994), as well as those that have not been scored against the IUCN criteria. Species and ecological systems are protected in private and municipal reserves, but currently most protected lands in the study area are managed by national governments.

**Results**

At the time of this study designated national protected areas accounted for 10.52% of the entire region (Table 2). Protected areas vary considerably in size, from 209 ha for Cavernas de Depechón to over 2.5 million hectares for Alto Purus. These protected areas are predominantly designated as national parks (7.5% of the region), including areas such as Manú or Noel Kempff Mercado. The next largest regional percentage (2.5%) is in areas designated as sustainable use zones, including portions of Pacaya Samíria, Tambopata, and others. Some 0.2% and 0.3% respectively include natural monuments, such as Machu Picchu, or managed natural habitats, such as in Manuripi. Currently, no areas within the study region are designated as either strict reserves or protected landscapes. Approximately 1.5% of the region includes areas proposed for conservation, but not yet officially designated as such. These include areas such as Pucacuro, Sierra del Divisor, and others.

**Ecological systems.**

Evaluating the protection of ecological systems, revealed that 10 ecosystem types have greater than 50% of their mapped distribution occurring within protected areas (the three left-hand columns combined). Unfortunately, for nearly half of the 91 ecosystem types in the study region (44 types), have less than 10% of their extent falling within protected areas; with 24 of these with no significant amount included (Table 3). These under-represented types encompass over 19 million hectares in aggregate, but only 1 million ha of these falls within protected areas. In this region, these types should clearly provide one focus of new conservation effort. Of these under-represented types and 21 are known only from Bolivia, 9 are found only in the Peruvian portion of the study region. Additional detailed results of the overlays of ecosystems and protected areas are found in Swenson et al. Table 5 and Appendix S5.

Table3. Number of types, total area (ha), area within protected areas, percent of the study region, and proportion of all protected ecosystem area within the Amazon Basin of Peru and Bolivia.

| Percent Area within Protected Areas | >90% | 75-90% | 50-74% | 25-49% | 10-24% | 1-9% | Zero/ Negligible |
| --- | --- | --- | --- | --- | --- | --- | --- |
| Number of Ecosystem Types | 3 | 3 | 3 | 17 | 22 | 21 | 24 |
| Total Area (hectares) | 611,204 | 74,543 | 246,838 | 37,055,561 | 57,968,634 | 11,868,675 | 7,433,773 |
| Total Area Protected (hectares) | 564,588 | 61,336 | 142,527 | 13,618,324 | 8,408,511 | 1,034,632 | 2,396 |
| Percent of Project Region Protected | 0.45% | 0.05% | 0.11% | 10.96% | 6.77% | 0.83% | 0.002% |
| Proportion of All Ecosystem Types within Protected Areas | 2.33% | 0.26% | 0.59% | 56.18% | 34.69% | 4.34% | 0.01% |

**References for Part III**

Ferrier, S., R.L. Pressey, and T.W. Barrett. 2000. A new predictor of the irreplaceability of areas for achieving a conservation goal, its application to real-world planning, and a research agenda for further refinement. Biological Conservation **93**:303-325.

McNeely, J.A., and K. Miller. 1984. National Parks, Conservation, and Development: The Role of Protected Areas in Sustaining Society, International Union for the Conservation of Nature. Smithsonian Institution Press.

Pressey, R. L., I. R. Johnson, and P. D. Wilson. 1994. Shades of irreplaceability: towards a measure of the contribution of sites to a reservation goal. Biodiversity and Conservation 3:242–262.

Pressey, R. L. 1999. Applications of irreplaceability analysis to planning and management problems. Parks 9:42–51.

Pressey, R.L., M. Watts, M. Ridges & T. Barrett. 2005. C-Plan conservation planning software. User Manual. New South Wales Department of Environment and Conservation, Australia.

Rissler, L.J., Hijmans, R.J., Graham, C.H., Moritz, C., and D.B. Wake. 2006. Phylogeographic Lineages and Species Comparisons in Conservation Analyses: A Case Study of California Herpetofauna. The American Naturalist 167: 655-666.

Slocum, T.A. 1999. Thematic cartography and visualization. Prentice Hall, New Jersey.

Young, B.E. 2007. Endemic species distributions on the east slope of the Andes in Peru and Bolivia. NatureServe, Arlington, Virginia. Available from <http://www.natureserve.org/andesamazon> (accessed 2007).

Young, B.E., I. Franke, P.A. Hernandez, S.K. Herzog, L. Paniagua, A. Soto, C. Tovar, and T. Valqui. 2009. Using spatial models to predict areas of endemism and gaps in the protection of Andean slope birds. Auk **126**:554-565.
