# Supplementary material for: Plant and animal endemism in the eastern Andean slope: challenges to conservation
Source: BMC Ecol. 2012 Jan 27;12:1. doi: 10.1186/1472-6785-12-1 (PMC3311091; doi:10.1186/1472-6785-12-1)
Supplement: Additional file 5 — Sources of species locality data and expert reviewer list. [file 1472-6785-12-1-S5.DOC]

**File 5. Sources of endemic species locality data and expert reviewer list**

I. Natural history museums that contributed animal locality data.

II. Herbaria that contributed plant locality data.

III. Literature sources of endemic species locality data.

IV. Reviewers of locality data and draft distribution maps.

V. Literature references used for species locality data

| **I. Natural history museums that contributed animal locality data.** | | |  |
| --- | --- | --- | --- |
|  | **Collection** | | |
| **Institution** | **Birds** | **Mammals** | **Amphibians** |
| American Natural History Museum | x | x | x |
| Associación Armonía | x |  |  |
| Carnegie Museum of Natural History | x | x |  |
| Centro de Biodiversidad y Genética (Cochabamba, Bolivia) |  | x |  |
| Centro de Datos para la Conservación (CDC) de la Universidad Nacional Agraria La Molina |  | x |  |
| Colección Boliviana de Fauna | x | x |  |
| Delaware Museum of Natural History | x |  |  |
| Field Museum | x | x | x |
| Louisiana State University, Museum of Natural Science | x | x | x |
| Museo de Historia Natural Noel Kempff Mercado (Santa Cruz, Bolivia) | x |  |  |
| Museo de Historia Natural, Universidad Nacional Mayor de San Marcos | x | x | x |
| The Academy of Natural Sciences | x |  |  |
| United States National Museum of Natural History | x | x | x |
| University of California, Berkeley, Museum of Vertebrate Zoology | x | x | x |
| University of Kansas, Museum of Natural History |  |  | x |
| University of Michigan, Museum of Zoology | x | x | x |
| University of New Mexico, Museum of Southwestern Biology | x | x |  |
| Yale Peabody Museum | x |  |  |

**II. Herbaria that contributed plant locality data.**

Albion R. Hodgdon Herbarium. University of New Hampshire. Durham, New Hampshire.

Bailey Hortorium Herbarium, Cornell University. Ithaca, New York.

Botanical Museum. Lund, Sweden.

Botanischer Garten und Botanisches Museum Berlin-Dahlem. Berlin, Germany

Conservatoire et Jardin botaniques de la Ville de Genève. Geneve, Switzerland.

Daubeny Herbarium. University of Oxford. Oxford,England, U.K.

Dudley Herbarium. California Academy of Sciences. San Francisco, California.

Field Museum of Natural History. Chicago, Illinois.

Fielding-Druce Herbarium. University of Oxford. Oxford, England, U.K.

Harvard University Herbaria. Cambridge, Massachusetts.

Herbario Amazonense. Universidad Nacional de la Amazónia Perúana. Iquitos, Perú

Herbario del Oriente Boliviano. Museo de Historia Natural Noel Kempff Mercado. Universidad Autónoma Gabriel René Moreno. Santa Cruz, Bolivia.

Herbario Nacional Colombiano. Universidad Nacional de Colombia. Bogota, Colombia.

Herbario Nacional de Bolivia. La Paz, Bolivia.

Herbario Nacional del Ecuador. Museo Ecuatoriano de Ciencias Naturales. Quito, Ecuador.

Herbario Nacional Forestal Martín Cárdenas. Cochabamba, Bolivia.

Herbario Nacional. Universidad Nacional Autónoma de México. Mexico City, Mexico.

Herbario Selva Central. Oxapampa, Perú.

Herbario Vargas. Universidad Nacional San Antonio Abad del Cusco. Cusco, Perú.

Herbario. Museo de Historia Natural, Universidad Nacional Mayor de San Marcos. Lima, Perú.

Herbario. Fundación Miguel Lillo. Tucuman, Argentina.

Herbario. Instituto de Botánica Darwinion. Buenos Aires, Argentina.

Herbario. Instituto de Botánica del Nordeste. Corrientes, Argentina.

Herbario. Instituto Nacional de Tecnología Agropecuaria. Buenos Aires, Argentina

Herbario. Proyecto BOLFOR. Santa Cruz, Bolivia.

Herbario. Real Jardín Botánico. Madrid, Spain.

Herbario. Universidad Nacional Agraria La Molina. Lima, Perú.

Herbario. Universidad Nacional de Cajamarca. Cajamarca, Perú.

Herbario. Universidad Nacional de Córdoba. Cordoba, Argentina.

Herbário. Universidade de São Paulo. São Paulo, Brazil.

Herbarium of the University of Arizona. Tucson, Arizona.

Herbarium Senckenbergianum. Forschungsinstitut Senckenberg. Frankfurt, Germany.

Herbarium Truxillense. Universidad Nacional de La Libertad-Trujillo. Trujillo, Perú.

Herbarium. Naturhistorisches Museum Wien. Vienna, Austria.

Herbarium. Pomona College. Claremont, California.

Herbarium. Royal Botanic Gardens, Kew. Richmond, England, U.K.

Herbarium. University of Michigan. Ann Arbor, Michigan.

Herbarium. Uppsala University. Uppsala, Sweden.

Herbarium. Academy of Natural Sciences. Philadelphia, Pennsylvania.

Herbarium. Albrecht-von-Haller-Institut für Pflanzenwissenschaften. Universität Göttingen. Göttingen, Germany.

Herbarium. California Academy of Sciences. San Francisco, California.

Herbarium. Heidelberger Institut für Pflanzenwissenschaften. Universität Heidelberg. Heidelberg, Germany.

Herbarium. Institut für Systematische Botanik. Universität Zürich. Zürich. Switzerland.

Herbarium. Marie Selby Botanical Gardens. Sarasota, Florida.

Herbarium. Missouri Botanical Garden. St. Louis, Missouri.

Herbarium. National Museum in Prague. Prague, Czech Republic.

Herbarium. Natural History Museum of Los Angeles County. Los Angeles, California.

Herbarium. Rancho Santa Ana Botanic Garden. Claremont, California.

Herbarium. Royal Botanic Garden. Edinburgh, Scotland, U.K.

Herbarium. Swedish Museum of Natural History. Stockholm, Sweden.

Herbarium. The Natural History Museum. London,England, U.K.

Herbarium. United States National Arboretum. Washington D.C.

Herbarium. University of California. Los Angeles, California.

Herbarium. University of Copenhagen. Copenhagen, Denmark.

Herbarium. University of Texas at Austin. Austin, Texas.

Herbarium. V. L. Komarov Botanical Institute. Saint Petersburg, Russia.

Herbier National de Paris. Muséum National d'Histoire Naturelle. Paris, France.

Nationaal Herbarium Nederland, Leiden University branch. Leiden, Netherlands

United States National Herbarium. Smithsonian Institution. Washington D.C.

University Herbarium. University of California. Berkeley, California.

University of Aarhus Herbarium Jutlandicum. Aarhus, Denmark.

William and Lynda Steere Herbarium. New York Botanical Garden. Bronx, New York.

**III. Reviewers of locality data and draft distribution maps.**

**Plants**

S. Altamirano

W. Anderson

C. Anderson

C. Antezana

S. Arrazola

S. Beck

G. Barriera

P. Berry

N. de la Barra

S. Dressler

W. Galiano

C. Hughes

D. Ibañez

S. Leiva

B. León

P.-A. Loizeau

J. Luteyn

R. Meneses

J. Mitchell

B. Mostacedo

G. Navarro

P. Nuñez

T. Pennington

G. Prance

V. Quipuscoa

E. Rodríguez

A. Sagastegui

I. Sánchez

B. Stein

A. Tupayachi

J. Wood

C. Zambrana

M. Zapata

**Amphibians**

C. Aguilar

W. Arízabal

J. C. Chaparro

J. Córdova

D. Embert

V. Morales

D. Neira

S. Reichle

L. Rodríguez

P. Venegas

**Mammals**

V. Pacheco

H. Quintana

R. Timm

J. Vargas

**Birds**

I. Franke

S. Herzog

D. Lane

J. O'Neill

T. Valqui

**IV. Literature sources of endemic species locality data**

**A) References used for bird locality data**

Alonso, J. A. and B. M. Whitney. 2001. A new *Zimmerius tyrannulet* (Aves: Tyrannidae) from white sand forests of Northern Amazonian Peru. Wilson Bulletin 113: 1-9

Alonso, J. A. and B. M. Whitney. 2003. New distributional records of birds from white-sand forests of the Northern Peruvian Amazon, with implications for biogeography of Northern South America. The Condor 105( 2): 552-566.

Angehr, G. R., C. Aucca, and D. G. Christian. 1998. Birds II. Pages 165-185 in Alonso-M., A. and F. Dallmeier. 1998. Biodiversity assessment of the Lower Urubamba Region, Peru. Cashiriari-3 Well Site and the Camisea and Urubamba Rivers. Washington, DC: Smithsonian Institution/MAB Biodiversity Program.

Aucca C. 1998. Birds I. Pages 143-163 in Alonso-M., A. and F. Dallmeier. 1998. Biodiversity assessment of the Lower Urubamba Region, Peru. Cashiriari-3 Well Site and the Camisea and Urubamba Rivers. Washington, DC: Smithsonian Institution/MAB Biodiversity Program.

Barnes, R., S. H. M. Butchart, C. W. N. Davies, M. Fernandez, and N. Seddon. 1997. New distributional information on eight bird species from northern Peru. Bulletin of the British Ornithologists' Club 117: 69-74.

Bates, J. M. and R. M. Zink. 1994. Evolution into the Andes: Molecular evidence for species relationships in the genus *Leptopogon*. Auk 111 (3). 507-515.

Bryce R., B. Hennessey, R. Macleod, K. L. Evans, S. R. Ewing, S. K. Herzog, A. Maccormick, and M. I. Gomez. 2005. First sound recordings, new behavioural and distributional records, and a review of the status of Scimitar-winged Piha *Lipaugus uropygialis*. Cotinga 24, 102-106.

Davis, T. J. 1986. Distribution and natural history of some birds from the departments of San Martin and Amazonas, northern Peru. Condor 88:50-56.

Davis, T. J. and J. P. O'Neill. 1986. A new species of antwren (Formicariidae: *Herpsilochmus*) from Peru, with comments on the systematics of other members of the genus. Wilson Bulletin 98:337-352.

Engblom, G., C. A. Chutas, G. F. Meza, E. Samochuallpa, and W. Palomino. 2002. The conservation of *Polylepis*-adapted birds at Abra Málaga, Cuzco, Peru. Cotinga 17:56-59.

Fitzpatrick, J. W. and J. P. O’Neill. 1986. *Otus petersoni*, a new Screech-Owl from the Eastern Andes, with systematic notes on *O. colombianus* and *O. ingens*. Wilson Bulletin 98: 1-14.

Fjeldså, J., N. Krabbe, and T. A. Parker. 1987. Rediscovery of *Cinclodes excelsior aricomae* and notes on the nominate race. Bull. Brit. Orn. Club 107: 112-114.

Fjeldså, J. and M. Kessler. 1996. Conserving the biological diversity of *Polylepis* woodlands of the highlands of Peru and Bolivia. Nordeco and Zoological Museum, Copenhagen.

Gerhart, N. G. 2004. Rediscovery of the Selva Cacique (*Cacicus koepckeae*) in southeastern Peru with notes on habitat, voice, and nest. Wilson Bulletin 116:74-82.

Gilardi, J. D. and C. A. Munn. 1998. Patterns of activity, flocking and habitat use in parrots of the Peruvian Amazon. Condor 100: 641-653.

Haffer, J. and J. W. Fitzpatrick. 1985. Geographic variation in some Amazonian forest birds. Ornithol. Monogr. 36:147-168.

Isler, M. L., J. Alvarez A., P. R. Isler, and B. M. Whitney. 2001. A new species of *Percnostola* antbird (Passeriformes: Thamnophilidae) from Amazonian Peru, and an analysis of species limits within *Percnostola rufifrons*. Wilson Bulletin 113:164-176.

Johnson, N.K. and R. E. Jones. 2001. A new species of tody-tyrant (Tyrannidae: *Poecilotriccus*) from northern Peru. The Auk 118(2):334-341.

Kratter, A. W. 1997. Bamboo specialization by Amazonian birds. Biotropica 29 (1):100-110.

Lane, D. F. and T. Pequeño. 2004. Apendix 6. Birds. Pages 274- 286 in C. Vriesendorp, L. Rivera Chávez, D. Moskovits, and J. Shopland (eds.), Perú: Megantoni, Rapid Biological Inventory 15, Field Museum of Natural History, Chicago, Illinois, USA.

Lane, D., T. Pequeño, and W. Flores. 2003. Appendix 5, Aves observadas en tres sitios en el río Yavarí, Perú, entre marzo y abril de 2003. Pages 254-267 in N. Pitman, C. Vriesendorp, D. Moskovits (Eds.), Perú : Yavarí. Rapid Biological Inventories Report 11. The Field Museum, Chicago, Illinois, USA.

Lane, D. F., H. T. Valqui, A. J. Alvarez, J. Armenta, and K. Eckhardt. 2006. The rediscovery and natural history of the White-masked Antbird (*Pithys castaneus*). Wilson Journal of Ornithology 118(1): 13-22.

Lloyd, H. 2004. Habitat and population estimates of some threatened lowland forest bird species in Tambopata, southeast Peru. Bird Conservation International 14:261-277.

Lloyd, H. and A. P. Marín. 2000. The lowland rainforest bird communities of five ecotourism locations in Tambopata, Madre de Dios, southeastern Peru. Tambopata Reserve Society, London, UK. Available at http://www.geocities.com/project_tambopata_peru/index.htm.

Lowery, G. H. Jr., and J. P. O'Neill. 1969. A new species of Antpitta from Peru and a revision of the subfamily Grallariinae. Auk 86:1-12.

Lowery, G. H. and. A. Tallman. 1976. A new genus and species of nine-primaried oscine of uncertain affinities from Peru. Auk 93: 415-428.

Mazar Barnett, J., G. M. Kirwan, and J. Minns. 2004. Neotropical notebook (other records received). Cotinga 21: 84-87.

Mee, A., J. Ohlson, I. Stewart, M. Wilson, P. Örn, and J. Diaz Ferreyra. 2002. The Cerros del Sira revisited: birds of submontane and montane forest. Cotinga 18:46-57.

O’Neill, J. P. and G. R. Graves. 1977. A new genus and species of owl (Aves: Strigidae) from Peru. Auk 94:409-416.

Parker, T. A. III. 1983. Rediscovery of the rufous-fronted antthrush (*Formicarius rufifrons*) in southeastern Peru. Gerfaut 73(3): 287-289.

Parker, T. A. III, and J. P. O’Neill. 1980. Notes on little known birds of the upper Urubamba Valley, southern Peru. Auk 97: 167-l 76.

Parker, T. A. III, P. K. Donahue, and T. S. Schulenberg. 1994. Appendix 3, Birds of the Tambopata Reserve (Explorer's Inn Reserve). Pages 106-124 in R. B. Foster et al. (Eds.), The Tambopata-Candamo Reserved Zone of southeastern Perú: a biological assessment. RAP Working papers 6, Conservation International, Washington, DC.

Pequeño, T., E. Salazar, and C. Aucca. 2001. Bird species observed at Llactahuaman (1710 m), southern Cordillera de Vilcabamaba, Peru. Pages 249-251 in L. E. Alonso, A. Alonso, T. S. Schulenberg, and F. Dallmeier (Eds.), Biological and social assessments of the Cordillera de Vilcabamba, Peru. RAP Working paper s 12 and SI/MAB Series 6, Conservation International, Washington, DC.

Pequeño, T., E. Salazar, and C. Aucca. 2001. Bird species observed at Wayrapata (2445 m), southern Cordillera de Vilcabamba, Peru. Pages 252-254 in L. E. Alonso, A. Alonso, T. S. Schulenberg, and F. Dallmeier (Eds.), Biological and social assessments of the Cordillera de Vilcabamba, Peru. RAP Working paper s 12 and SI/MAB Series 6, Conservation International, Washington, DC. `

Remsen, J. V. Jr. 1981. Community ecology of Neotropical kingfishers. Univ. Calif. Publ. Zool. 124: 1-128.

Remsen, J. V. Jr. 1984. Natural history notes on some poorly known Bolivian birds. Part 2. Gerfaut 74: 163-l 79.

Remsen, J. V. Jr. 1993. Zoogeography and geographic variation in *Atlapetes rufinucha* (Aves: Emberizinae), including a distinctive new subspecies, in southern Peru and Bolivia. Proc. Biol. Soc. Wash. 106: 429-435.

Remsen, J.V. Jr. 1997. Studies in Neotropical ornithology honoring Ted Parker. Ornithol. Monograph 48, A.O.U., Washington, D.C.

Robbins, M. B., G. H. Rosenberg, and F. Sornoza Molina. 1994. A new species of cotinga (Cotingidae: *Doliornis*) from the Ecuadorian Andes, with comments on plumage sequences in *Doliornis* and *Ampelion*. Auk 111: 1-7.

Schifter, H. 2000. A further specimen of the Fine-barred Piculet (*Picumnus subtilis*) in the Museum of Natural History, Vienna, Austria. Orn. Neotrop. 11: 247-248.

Schulenberg, T. S. and G. Graham. 1981. A new subspecies of *Anairetes agraphia* (Tyrannidae) from northern Peru. Bulletin of British Ornithologists’ Club 101(1): 241-243.

Schulenberg, T. S. and M. D. Williams. 1982. A new species of antpitta (*Grallaria*) from northern Peru. Wilson Bulletin. 94:105-113.

Schulenberg, T. S. and L. C. Binford. 1985. A new species of tanager (Emberizidae, Thraupinae, *Tangara*) from southern Peru. Wils. Bull. 97: 413-420.

Schulenberg, T. S. and T. A. Parker. 1997. Notes on the Yellow-browed Toucanet *Aulacorhynchus huallagae*. Orn. Monogr. 48: 717-720.

Schulenberg, T. S., L. López, G. Servat, and A. Valdés. 2001. Appendix 13, Preliminary list of the birds at three sites in the northern Cordillera de Vilcabamba, Peru. Pages 241-248 in L. E. Alonso, A. Alonso, T. S. Schulenberg, and F. Dallmeier (Eds.), Biological and social assessments of the Cordillera de Vilcabamba, Peru. RAP Working paper s 12 and SI/MAB Series 6, Conservation International, Washington, DC.

Schulenberg, T. S., J. P. O'Neill, D. F. Lane, T. Valqui, and C. Albújar. 2001. Appendix 5, Bird species recorded in three watersheds of the northern Cordillera Azul and along the Río Cushabatay, Depto. Loreto, Peru, 1996-2000. Pages 206-225 in W. S. Alverson, L.O.

Terborgh, J. 1971. Distribution on environmental gradients: Theory and a preliminary interpretation of distributional patterns in the avifauna of the Cordillera Vilcabamba, Peru. Ecology 52(1): 23-40.

Tobias, J. 2003. Further sightings of Selva Cacique *Cacicus koepckeae* in Manu National Park, Peru. Cotinga 19:79-80.

Valqui, T. and J. Fjeldså. 1999. New brush-finch *Atlapetes* from Peru. Ibis 141:194-198.

Véliz, C. 2003. Reporte de los trabajos realizados y los registros existentes para la flora y fauna del Bosque de Protección Alto Mayo. Parkswatch Peru 33 pp.

Vogel, C. J. and S. E. Davis. 2002. A new site for Royal Cinclodes *Cinclodes aricomae* and other noteworthy records from the Ilampu Valley, Bolivia. Cotinga 18: 104-106.

Weske, J. S. and J. W. Terborgh. 1974. *Hemispingus parodii*, a new species of tanager from Peru. Wilson Bull. 86:97-103

Weske, J. S. and J. W. Terborgh. 1981. *Otus marshalli*, a new species of screech-owl from Peru. Auk 98(1): l-7.

Whitney, B. M. 1994. A new *Scytalopus* Tapaculo (Rhinocryptidae) from Bolivia, with notes on other Bolivian members of the genus and the *magellanicus* complex. The Wilson Journal of Ornithology 106(4): 585-8

Whitney, B. M. and J. A. Alonso. 1998. A new *Herpsilochmus* antwren (Aves: Thamnophilidae) from northern Amazonian Peru and adjacent Ecuador: the role of edaphic hetergeneity of terra firme forest. Auk 115: 559-576.

Zimmer, J. T. 1939. Studies of Peruvian birds. XXXII. The genus *Scytalopus*. Amer. Mus. Novit. 1044:1-18.

**B) References used for mammal locality data**

Anderson, S. 1997. Mammals of Bolivia, taxonomy and distribution. Bulletin of the American Museum of Natural History 231:1-652.

Butchart, S. H. M, R. Barnes, C. W. N. Davies, M. Fernandez, and N. Seddon. 1995. Threatened mammals of the Cordillera de Colan, Peru. Oryx 29(4): 275-281.

Eisenberg, J. and K. Redford. 1999. Mammals of the Neotropics. Vol 3. The central Neotropics: Ecuador, Peru, Bolivia, Brazil. University of Chicago Press, Chicago.

Emmons, L. H., C. Ascorra, and M. Romo. 1994. Mammals of the Río Heath and Peruvian pampas. RAP Working Papers, 6:69-71, 146-149.

Felton, A., A. M. Felton, R. B. Wallace and H. Gomez. 2006. Identification, behavioral observations, and notes on the distribution of the titi monkeys *Callicebus modestus* Lönnberg, 1939 and *Callicebus olallae*, Lönnberg 1939. Primate Conservation 2006 (20): 41-46.

Glanz, W. and S. Anderson. 1990. Notes on Bolivian mammals 7. A new species of *Abrocoma* (Rodentia) and relationships of the Abrocomidae. American Museum Novitates:1-32.

Hinojosa, F., S. Anderson, and J. L. Patton. 1987. Two new species of *Oxymycterus* (Rodentia) from Peru and Bolivia. American Museum Novitates 2898:1-17.

Malygin, V.M., V.M. Aniskin, S. I. Isaev and A. N. Milishnikov. 1994. *Amphinectomys savamis* Malygin Gen. et sp. n., a new genus and a new species of water rat (Cricetidae, Rodentia) from Peruvian Amazonia. Zoologicheskii Zhurnal, 73:195-208.

Mark, M. 2003. Some observations on *Callicebus oenanthe* in the Upper Mayo Valley, Peru. Neotropical Primates 11(3):183-187.

Musser, G., and M. Carleton. 2005. Superfamily Muroidea, in Mammal species of the world, a taxonomic and geographic reference 3rd edition, vol 2. ed. Wilson, D. and D. Reeder. Baltimore: The Johns Hopkins University Press.

Myers, P. and J. L. Patton. 1989. The *Akodon* of Peru and Bolivia - revision of the *fumeus* group (Rodentia: Sigmodontinade). Occ. Pap. Mus. Zool., Univ. Michigan, 721: 1-35.

Oliveira, J.A. 1998. Morphometric assessment of species groups in the South American rodent genus *Oxymycterus* (Sigmodontinae), with taxonomic notes based on the analysis of type material. Ph.D. dissertation, Texas Tech University, Lubbock, Texas, 320 pp.

Pacheco, V. 2003. Phylogenetic analyses of the Thomasomyini (Muroidea: Sigmodontinae) based on morphological data. Ph.D. dissertation. The City University New York, New York, USA.

Pacheco, V., S. Solari, and P. M. Velazco. 2004. A new species of *Carollia* (Chiroptera: Phyllostomidae) from the Andes of Peru and Bolivia. Occasional Papers, Museum of Texas Tech University 236: 1-15.

Patton, J.L., and M. F. Smith. 1992. mtDNA phylogeny of Andean mice: a test of diversification across ecological gradients. Evolution 46:174-183.

Rumiz, D. I., E. Pardo, C. F. Eulert, R. Arispe, R. B.Wallace, H. Gomez, and B. Rios-Uzeda. In press. New records and a status assessment of a rare dwarf brocket deer from the montane forests of Bolivia. Journal of Zoology.

van Roosmalen, M G. M., T. van Roosmalen, and R. A. Mittermeier. 2002. A taxonomic review of the titi monkeys, genus *Callicebus* Thomas, 1903, with the description of two new species, *Callicebus bernhardi* and *Callicebus stephennashi*. Brazilian Amazonia. Neotropical Primates 10: 1-52.

Salazar-Bravo, J., E. Yensen, T. Tarifa, and T. L. Yates. 2002. Distributional records of Bolivian mammals. Mastozoologia Neotropical 9:70-78.

Salazar-Bravo, J., T. Tarifa, L. F. Aguirre, E. Yensen, and T. L. Yates. 2003. Revised checklist of Bolivian mammals. Occasional Papers Museum of Texas Tech University 220: 1-27.

Thomas, O. 1901. New mammals from Peru and Bolivia, with a list of those recorded from the Inambari River, upper Madre de Dios. Ann. Mag. Nat. Hist. 7(5): 148-153.

Thomas, O. and J. St. Leger. 1926. The Godman-Thomas expedition to Peru. IV. On mammals collected by Mr. R.W. Hendee north of Chachapoyas, Province of Amazonas, north Peru. Annals and Magazine of Natural History, ser. 9, 18:345-349.

Voss, R. S., T. Tarifa, and E. Yensen. 2004. An introduction to *Marmosops* (Marsupialia: Didelphidae), with the description of a new species from Bolivia and notes on the taxonomy and distribution of other Bolivian forms. American Museum Novitates 3466, 40 pp.

Wallace, R. B., H. Gómez, A. Felton, and A. M. Felton. 2006. On a new species of titi monkey, genus *Callicebus* Thomas (Primates, Pitheciidae), from western Bolivia with preliminary notes on distribution and abundance. Primate Conservation 20: 29-39.

Woods, C. and W. Kilpatrick. 2005. Infraorder Hystricognathi, in Mammal species of the world, a taxonomic and geographic reference, Vol 2. 3rd edition, ed. Wilson, D. and D. Reeder. Baltimore, The Johns Hopkins University Press.

**C) References used for Amphibian locality data**

Aguayo, V., R. Cidar, and M. Harvey. 2001. Dos nuevas especies de *Phrynopus* (Anura: Leptodactylidae) de los bosques nublados de Bolivia. Rev. Biol. Trop. 49(1):333-345.

Aichinger, M. 1991. A new species of poison-dart frog (Anura, Dendrobatidae) from the Serrania de Sira, Peru. Herpetologica 47:1-8.

Barrio-Amorós, C. L. and D. R. Neira. 2004. Geographic distribution: *Hyla joannae*. Herpetological Review 35(4):404.

Boulenger, G. 1918. *Hylella ocellata*. Ann. Mag. Nat. Hist. 9( 2): 433.

Cannatella, D. C. 1982. Leaf-frogs of the *Phyllomedusa perinesos* group Anura: Hylidae). Copeia (3):501–513.

Cannatella, D. C. and W. E. Duellman. 1982. Two new species of *Centrolenella*, with a brief review of the genus in Peru and Bolivia. Herpetologica 38(3):380–388.

De la Riva, I. 1994. A new aquatic frog of the genus *Telmatobius* (Anura, Leptodactylidae) from Bolivian cloud forests. Herpetologica 50(1):38-45.

De la Riva, I. and J. D. Lynch. 1997. New species of *Eleutherodactylus* from Bolivia (Amphibia: Leptodactylidae). Copeia 1997 (1): 151-157.

De la Riva, I. and M. Harvey. 2003. A new species of *Telmatobius* from Bolivia and redescription of *T. simonsi* Parker, 1940 (Amphibia: Anura: Leptodactylidae). Herpetologica 59(1):127-142.

De la Riva, I. and J. C. Chaparro. 2005. A new species of *Hyla* from cloud forests of southeastern Peru (Anura: Hylidae). Amphibia-Reptilia 26 (4): 515-521.

De la Riva, I., A. James, and J. N. Rios. 2005. New species of *Telmatobius* (Anura: Leptodactylidae) from humid paramo of Peru and Bolivia. Journal of Herpetology 39 (3):409-416.

Duellman, W. E. 1978. Three new species of *Eleutherodactylus* from Amazonian Perú (Amphibia: Anura: Leptodactylidae). Herpetologica 34:264-270.

Duellman, W. E. 1982. A new species of small yellow *Hyla* from Peru (Anura: Hylidae). Amphibia-Reptilia 3:153-160.

Duellman, W. E. 1987. Two new species of marsupial frogs (Anura: Hylidae) from Peru. Copeia 1987:903-909

Duellman, W. E. 1991. A new species of Leptodactylid frog, genus *Phyllonastes*, from Peru. Herpetologica. 47:9-13.

Duellman, W. E. 2000. Leptodactylid frogs of the genus *Phrynopus* in northern Peru with descriptions of three new species. Herpetologica 56(3):273-285.

Duellman, W. E. and L. Trueb. 1988. Cryptic species of hylid marsupial frogs in Peru. Journal of Herpetology. 22:159–179.

Duellman, W. E. and L. Trueb. 1989. Two new treefrogs of the *Hyla parviceps* group from the Amazon basin in southern Peru. Herpetologica 45:1-10.

Duellman, W. E. and R. Schulte. 1992. Description of a new species of *Bufo* from northern Peru with comments on phenetic groups of South American toads (Anura: Bufonidae). Copeia 1992(1): 162-172.

Duellman, W. E., I. De la Riva, and E. R. Wild. 1997. Frogs of the *Hyla armata* and *Hyla pulchella* groups in the Andes of South America, with definitions and analyses of phylogenetic relationships of Andean groups of Hyla. Scientific Papers Natural History Museum University of Kansas September 3:1-41.

Duellman, W. E. and I. De la Riva. 1999. Rediscovery and taxonomic status of *Hyla splendens* Schmidt, 1857 (Anura: Hylidae). Copeia 1999(1):197-199.

Duellman, W. E., E. Lehr, D. Rodríguez, and R. von May. 2004. Two new species of marsupial frogs (Anura: Hylidae: *Gastrotheca*) from the Cordillera Oriental in northern Peru. Scientific Papers, Natural History Museum, University of Kansas, No. 32, pp. 1–10.

Duellman, W. E. and P. Venegas. 2005. Marsupial frogs (Anura: Hylidae: *Gastrotheca*) from the Andes of northern Peru with descriptions of two new species. Herpetologica. 61:295-307.

Duellman W. E., E. Lehr, and P. J. Venegas. 2006. New species of *Eleutherodactylus* (Anura: Leptodactylidae) from the Andes of Northern Peru. Zootaxa. 1285: 51–64.

Dwyer, C. 1995. A new species of *Eleutherodactylus* from Peru (Anura: Leptodactylidae). Amphibia-Reptilia 16(3):245-256.

Gonzales L., S. Lötters, and S. Reichle. 1999. On the dendrobatid frogs from Bolivia: rediscovery of *Epipedobates bolivianus* (Boulenger, 1902), first record of *Colostethus brunneus* (Cope, 1887) and comments on other species (Anura: Dendrobatidae). Herpetozoa 12 (3/4) 179-186.

Grant, T. and L. Rodríguez. 2001. Two new species of frogs of the genus *Colostethus* (Dendrobatidae) from Peru and a redescription of *C. trilineatus* (Boulenger, 1883). Amer. Museum Novitates 3355:1-24.

Graybeal, A. and D. C. Cannatella. 1995. A new taxon of Bufonidae from Peru, with descriptions of two new species and a review of the phylogenetic status of supraspecific bufonid taxa. Herpetologica 51(2):105–131.

Harvey, M. and E. N. Smith. 1993. A new species of aquatic *Bufo* (Anura: Bufonidae) from cloud forests in the Serranía de Siberia, Bolivia. Proceedings of the Biological Society of Washington 106(3):442-449.

Hedges, S. B. 1990. A new species of *Phrynopus* (Anura, Leptodactylidae) from Peru. Copeia 1990:108-112.

Heyer, W. R., J. M. Garcia-Lopez, and A. J. Cardoso. 1996. Advertisement call variation in the *Leptodactylus mystaceus* species complex (Amphibia: Leptodactylidae) with a description of a new sibling species. Amphibia-Reptilia 17: 7-31.

Jungfer, K. H. and E. Lehr 2001. A new species of *Osteocephalus* with bicoloured iris from Pozuzo ( Peru: Departamento de Pasco) (Amphibia: Anura: Hylidae). Zoologische Abhandlungen Museum für Tierkunde Dresden 51:321 – 329.

Köhler, J. 2000. New species of *Eleutherodactylus* (Anura: Leptodactylidae) from cloud forest of Bolivia. Copeia 2000(2):516-520.

Köhler, J. and K.-H. Jungfer.1995: Eine neue Art und ein Erstnachweis von Fröschen der Gattung *Eleutherodactylus* aus Bolivien. Salamandra 31(3):149-156.

Köhler, J., V. R. Morales, S. Lötter, S. Reiche, and J. Aparicio. 1998. A new green species of frog, genus *Eleutherodactylus*, from Bolivia and Perú (Amphibia, Anura, Leptodactylidae). Stud. Neotrop. Fauna Environ. 33(3):93-99.

Köhler, J. and S. Reichle. 1998. Geographic distribution: Anura: *Cochranella pluvialis*. Hepetological Review 29(3):172.

Köhler, J. and S. Lötters. 1999 "1998". Annotated list of amphibian records from the Departamento Pando, Bolivia, with description of some advertisement calls. Bonner zoologische Beiträge 48 (3/4): 259-273.

Kohler, J. and S. Lötters. 1999. New species of the *Eleutherodactylus unistrigatus* group (Amphibia : Anura : Leptodactylidae) from montane rain forest of Bolivia Copeia 1999 (2): 422–427.

Köhler J. and S. Lötters. 2001. Description of small tree frog, genus *Hyla* (Anura: Hylidae), from humid Andean slopes of Bolivia. Salamandra 37 (3): 175-184.

Lavilla, E. O. and I. De la Riva. 1993. The larva of *Telmatobius bolivianus* (Anura, Leptodactylidae). Alytes 11 (2): 37-46

Lavilla, E. O., R. O. de Sa, and I. De la Riva. 1997. The tadpole of *Atelopus tricolor* (Anura: Bufinidae). Journal of Herpetology. 31:121-124.

Lehr, E. 2005. A new species of the *Eleutherodactylus nigrovittatus* group (Anura: Leptodactylidae) from Andean Peru. Herpetologica. 61(2):199-208.

Lehr, E., D. Rodríguez, and J. H. Córdova. 2002. A new species of *Phrynopus* (Amphibia, Anura, Leptodactylidae) from the Cordillera de Carpish (Departamento de Huánuco, Perú). Zool. Abh. 52:65-70.

Lehr, E., C. Aguilar, and M. Lundberg. 2004. A new species of *Phyllonastes* (Anura: Leptodactylidae) from Peru. Journal of Herpetology, 38, 214–218

Lehr, E., C. Aguilar, and W. E. Duellman. 2004. A striking new species of *Eleutherodactylus* from Andean Peru (Anura: Leptodactylidae). Herpetologica, Vol. 60, No. 2, pp. 275–280.

Lötters, S. and J. Köhler. 2000. A new toad of the *Bufo typhonius* complex from humid montane forests of Bolivia (Amphibia, Anura, Bufonidae). Spixiana 23(3):293-303.

Lötters, S., W. Haas, S. Schick, and W. Böhme. 2002. On the systematics of the harlequin frogs (Amphibia: Bufonidae: Atelopus) from Amazonia I: Description of a new species from the Cordillera Azul, Peru. Salamandra 38:95-104.

Lötters, S., R. Schulte, and W. E. Duellman. 2004. A new and critically endangered species of *Atelopus* from the Andes of northern Peru (Anura: Bufonidae). Rev. Esp. Herpetol. 18:101-109.

Márquez, R., I. De la Riva, and J. Bosch. 1996. Advertisement calls of three glass frogs from the Andean forests (Amphibia: Anura: Centrolenidae). The Herpetological Journal 6(3): 97-99.

Morales, V. R. 1992. Dos especies nuevas de *Dendrobates* (Anura, Dendrobatidae) para el Perú. Carib. J. SC. 28(2/3):191-199.

Morales, V. R. and R. Schulte. 1993. Dos especies nuevas de *Colostethus* (Anura, Dendrobatidae) en las vertientes de la Cordillera Oriental del Perú y Ecuador. Alytes 11(3): 97-106.

Morales, V. R. and J. Icochea. 2000. Review of the type material of *Eleutherodactylus mendax* and a new record of *Eleutherodactylus bromeliaceus* from Peru. J. Herpetol. 34: 158-160.

Myers, C. W. 1982. Spotted poison frogs: Descriptions of three new *Dendrobates* from western Amazonia, and resurrection of a lost species from "Chiriqui". American Museum Novitates. 2721:1-23.

Myers, C. W. and P. A. Burrowes. 1987. A new poison frog (*Dendrobates*) from Andean Colombia, with notes on a lowland relative. Amer. Museum Novitates (2899):1-17.

Myers, C., L. Rodríguez, and J. Icochea. 1998. *Epipedobates simulans*, a new cryptic species of poison frog from southeastern Peru, with notes on *E. macero* and *E. petersi* (Dendrobatidae). Amer. Museum Novitates, 3238:1-20.

Padial J. M., S. Reichle, and I. De la Riva. 2005. New species of *Ischnocnema (Anura: Leptodactylidae)* from the Andes of Bolivia. Journal of Herpetology 39(2):186-191.

Reichle, S. and J. Köhler. 1996. Geographic distribution: Gymnophiona: *Caecilia marcusi*. Herpetological Review 27(4):208.

Reynolds, R. P. and M. S. Foster. 1992. Four new species of frogs and one new species of snake from the Chapare region of Bolivia, with notes on other species. Herpetol. Monogr. 6:83–104.

Rivero, J. A. 1991. New *Colostethus* (Amphibia, Dendrobatidae) from South America. Breviora 493: 1–28.

Rivero, J.A. 1991. New Ecuadorean *Colostethus* (Amphibia, Dendrobatidae) in the collection of the National Museum of Natural History, Smithsonian Institution. Caribbean Journal of Science. 27(1-2):1-22.

Rodríguez, L. and C. W. Myers. 1993. A new poison frog from Manu National Park, southeastern Peru (Dendrobatidae, *Epipedobates*). Amer. Museum Novitates, 3068:1-15.

Schulte, R. 1999. Poison Dart Frogs. Vol. II. Peru: Species accounts. INIBICO.

Venegas, P. 2005. Geographic Distribution: *Phrynopus carpish*. Herpetological Review 36(1): 76.

Wiens, J. J. 1993. Systematics of the leptodactylid frog genus *Telmatobius* in the Andes of northern Peru. Occasional Papers of the Museum of Natural History University of Kansas 161:1–76.

Wild, E. R. 1995. New genus and species of Amazonian microhylid frog with phylogenetic analysis of New World genera. Copeia 1995: 837–849.
