# Supplementary material for: Plant and animal endemism in the eastern Andean slope: challenges to conservation
Source: BMC Ecol. 2012 Jan 27;12:1. doi: 10.1186/1472-6785-12-1 (PMC3311091; doi:10.1186/1472-6785-12-1)

**Additional File 6. Enlarged map of area and data.**

Fig 1. Enlarged area of southern Peru. Vertebrate species richness and ecological systems shown along the new interoceanic highway.


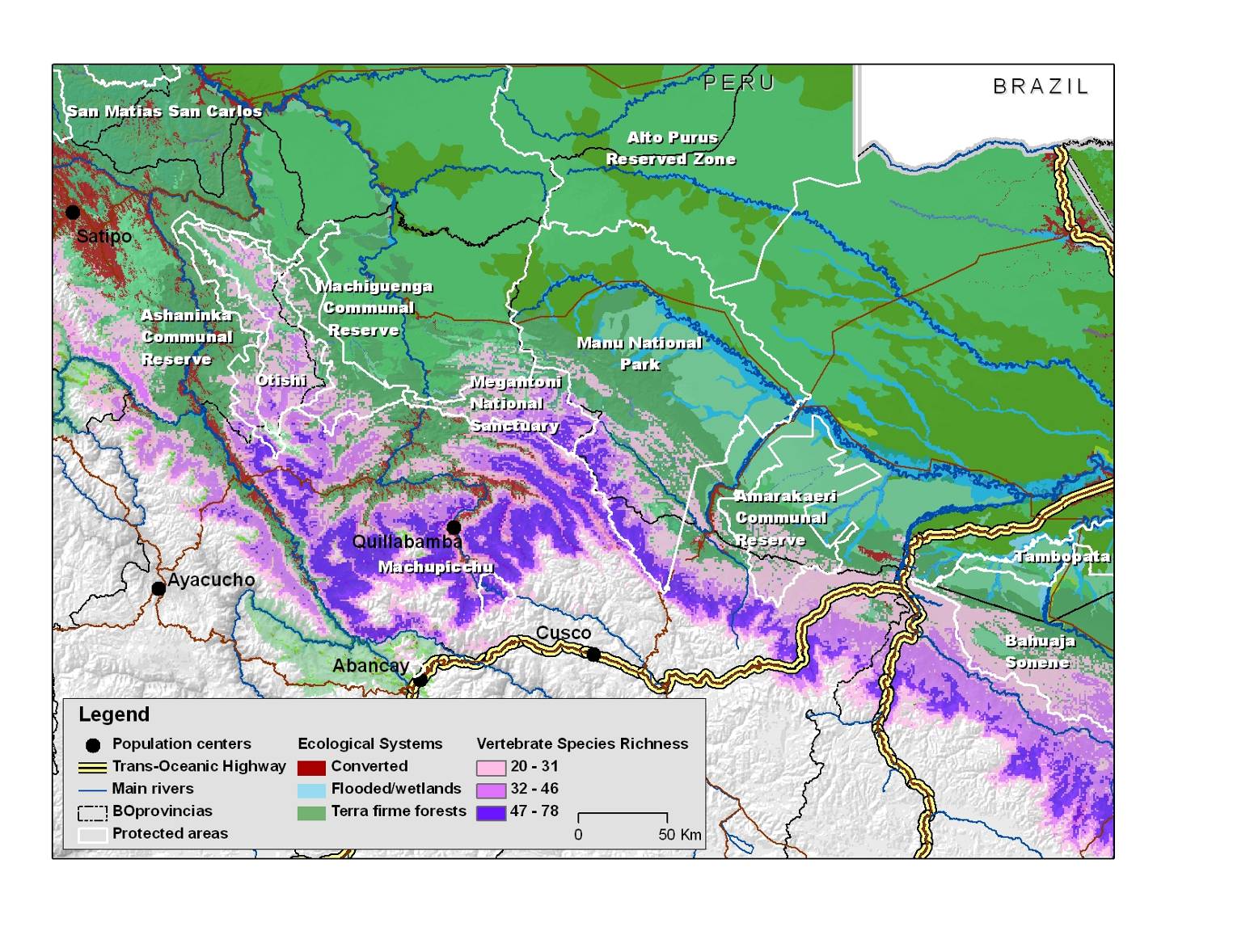

Supplement: Additional file 6 — Enlarged map of SE Peru; Vertebrate Endemism & Ecological Systems. [file 1472-6785-12-1-S6.DOCX]
